# ﻿Taxonomic and ecological remarks on *Solenopsisbivonae* species complex (Campanulaceae)

**DOI:** 10.3897/phytokeys.229.104324

**Published:** 2023-07-13

**Authors:** Salvatore Brullo, Cristian Brullo, Salvatore Cambria, Valeria Tomaselli, Alessandro Crisafulli, Giuseppe Siracusa, Pietro Minissale, Gianpietro Giusso del Galdo

**Affiliations:** 1 Dipartimento di Scienze Biologiche, Geologiche ed Ambientali, Università di Catania, via A. Longo 19, Catania, Italy Università di Catania Catania Italy; 2 Dipartimento di Biologia, Università di Bari Aldo Moro, via Orabona 4, 70125 Bari, Italy Università di Bari Aldo Moro Bari Italy; 3 Dipartimento ChiBioFarAm, Università degli Studi di Messina, Via Stagno d’Alcontres, 98100 Messina, Italy Università degli Studi di Messina Messina Italy

**Keywords:** ecology, Lobelioideae, Mediterranean flora, *Solenopsis*, taxonomy

## Abstract

The populations usually attributed to *Solenopsisbivonae* (Tineo) M.B.Crespo, Serra & A.Juan are investigated from a taxonomical and morphological viewpoint. Within this species complex, four new subspecies occurring in Sicily and Calabria are recognized, such as subsp. bivonae, subsp. madoniarum, subsp. peloritana and subsp. brutia. In addition, a new species from Cyprus described as *S.meikleana* and *S.bacchettae* from Sardinia must be included in this group. The synonymy, typification, description, seed testa morphology, chorology, ecology, illustrations, conservation status, and examined specimens for each taxon are provided. Besides, the analytical keys, distribution maps, and phytosociological arrangement regarding these taxa are given too.

## ﻿Introduction

*Solenopsis* C. Presl is a very peculiar genus of Campanulaceae, belonging to subfam. Lobelioideae, distributed in the Mediterranean and Macaronesian territories. Within this genus, two well-distinct groups can be recognized, which differ in habit and in flower structure (Crespo et al. 1998; Brullo et al. 2023a, b). The first one is characterized by a caulescent or subcaulescent habit with leaves all inserted on the scape and flowers with corolla provided by lobes slightly divaricated at the top. Conversely, the second one shows a stemless habit with leaves arranged in basal rosette and flowers with corolla provided by lobes markedly patent at the top. The only exception is represented by a species showing intermediate characters between the two groups since it has an erect scapose habit and flower corolla with clearly divaricated lobes at the top. The first group includes only annual species, such as *Solenopsislaurentia* C. Presl, widespread in the Mediterranean area and the Canary Islands, represented by several subspecies examined by Brullo et al. (2023a), to which *S.mothiana* C.Brullo, Brullo & Giusso, showing a punctiform distribution in Sicily (Isola Grande dello Stagnone), must be added. According to Brullo et al. (2023b), the second group includes many more species, such as *S.bivonae* (Tineo) M.B. Crespo et al. from Sicily and South Italy, and recorded also from Cyprus, *S.bacchettae* Brullo et al. from Sardinia, *S.minuta* C. Presl from Crete, *S.balearica* (E.Wimm.) Aldasoro et al. from Majorca, *S.corsica* (Meikle) M.B.Crespo et al. from Corse and N. Sardinia and *S.antiphonitis* Hadjik. & Hand from N. Cyprus. Recently, *S.minuta* has been observed in Cyprus by Christodoulou et al. (2020). Besides, *S.bicolor* (Batt.) Greuter & Burdet must be mentioned, occurring in Tunisia and Algeria, which is characterized by intermediate features between the two groups (Crespo et al. 1998). In the frame of taxonomic research on this genus, the populations currently attributed to *S.bivonae* (Tineo) M.B.Crespo, Serra & Juan are here investigated. In particular, this study of living material from several Mediterranean localities (Sicily, South Italy, Sardinia and Cyprus), and cultivated plants in the Botanical Garden of Catania emphasized the close morphological relationships among them. These investigations show that *S.bivonae* must be considered a species–complex, within which several morphologically well-distinct taxa can be identified. In order to verify the realistic distribution of these taxa, several herbarium materials were examined from all localities where the populations of this species were previously recorded. In particular, according to literature data (Pignatti 1982; Crespo et al. 1998; Brullo and Guarino 2018; Cambria et al. 2019), *S.bivonae* s.l. occurs in a scattered way in various Mediterranean territories, such as Sicily, South Italy, Sardinia, and Cyprus. As concerns the Sardinian populations, previously attributed to *S.bivonae* (Crespo et al. 1998), they have been described by Brullo et al. (2023b) as *S.bacchettae*, species well differentiated from the populations growing in Sicily, where occur *S.bivonae* s.s., described by Tineo (1827) as *Laurentiabivonae* on material collected along the Oreto River near Palermo, which represents its *locus classicus*. In particular, *S.bacchettae* differs from *S.bivonae* s.s. apart from some relevant features (hairy leaves, larger flowers, different coloured corolla, ultrastructure of pollen grains, and testa seed), also from the ecological point of view. In fact, *S.bacchettae* occurs prevalently along the small streams with flowing waters, while *S.bivonae* s.s. is localized on dripping walls or peat bogs. As regards the other populations of *S.bivonae*, significant morphological differences were observed in the individuals occurring in Sicily, South Italy and Cyprus, which allow for them to be treated as distinct taxa. Based on literature and herbarium data, *S.bivonae* in Sicily was recorded in many more stands than where it occurs today. Effectively, the populations of this species are linked to wet and very specialized natural habitats (dripping walls), many of which have now completely disappeared due to anthropic pressure. Currently, as proved by personal surveys, this species is still present in the locus classicus, where it has now become extremely rare due to pollution factors, as well as in other Sicilian stands. Other small populations of this species occur also along the Sosio river (Chiusa Sclafani), Ficuzza and in various localities of the Madonie massif, confirming previous literature data (Bivona-Bernardi 1806; Tineo 1827; Gussone 1843; Strobl 1883; Lojacono Pojero 1903; Marcenò et al. 1985; Gianguzzi et al. 2004; Giardina et al. 2007). Recently, a new population was observed by Cambria et al. (2020) near Vallone Pirtuso (Peloritani Mountains), as well as at Monte Canalotto near Piazza Armerina (unpublished record). As concerns the Sicilian populations, three taxa treated as distinct subspecies can be distinguished. Significant morphological features allow to differentiate these taxa, which show a well circumscribed distribution and peculiar ecological requirements. In particular, the populations of type subspecies (subsp. bivonae) occur at low altitudes, from sea level up to ca. 250 m a.s.l. (Oreto and Sosio rivers), while a second new subspecies (subsp. madoniarum) is widespread in the Madonie massif and in a small isolated stand near Piazza Armerina, where it grows at an altitude of 700–1600 m a.s.l. Finally, the third new subspecies (subsp. peloritana) is localized in a punctiform mountain locality of the Peloritani range at an elevation of 600–700 m a.s.l. The only continental populations of *S.bivonae* s.l. occur in North Calabria (S Italy), where Longo (1893, 1902) collected it along the banks of the Lao River near Laino Castello and Laino Borgo, while later Peruzzi and Gargano (2003) recorded it always along the Lao River, but below the village of Papasidero. From the taxonomical point of view, the Calabrian plants are clearly distinct from the other subspecies occurring in Sicily. Therefore, they are treated as a new subspecies of *S.bivonae* (subsp. brutia). Regarding the Cyprus populations, they were attributed by several authors (Poech 1842; Kotschy 1862; Unger and Kotschy 1865; Boissier 1875; Holmboe 1914) to *Laurentiatenella* DC., while Lindberg (1946) and Osorio–Tafall and Seraphim (1973) referred them to *Laurentiaminuta* (L.) DC. Finally, these populations were identified by Crespo et al. (1998) and Christodoulou et al. (2020) as *Solenopsisbivonae*. Wimmer (1948), in his revision of Lebelioideae, recognized three sections within the genus *Laurentia* Adans., and in particular he included the taxa previously attributed to the genus Solenopsis in the sect. Solenopsis (C. Presl) Hendl. Besides, this author referred the Cyprus populations to Laurentiaminuta(L.)DC.f.nobilis, quoting this taxon also from Palermo in Sicily, without the indication of the nomenclatural type. Afterward, Meikle (1979) used the Wimmer’s name, transferring this taxon to the genus *Solenopsis* and treating it as a subspecies, proposing the new combination S.minutasubsp.nobilis (F.E.Wimmer) Meikle, designating as lectotype a specimen collected by Kotschy in Cyprus (W). Moreover, Meikle (1979, 1985) mentioned this taxon apart from Cyprus, also in Sicily, emphasizing, however, that the Sicilian populations are quite variable, while its presence in Sardinia remains doubtful. Based on our morphological investigations, the populations of Cyprus are clearly differentiated from those present in Sicily and Calabria and can be attributed to a distinct species, described as *S.meikleana*, which is usually linked to dripping wet walls or river banks.

## ﻿Material and methods

The morphological investigations were conducted on wild plants collected in several Mediterranean territories (Sicily, South Italy, Sardinia, and Cyprus) and cultivated in the Botanical Garden of Catania (Italy). The morphological features were analyzed based on at least twenty individuals for each examined population, with well-developed vegetative and reproductive structures. The living material was observed under a Zeiss Stemi SV 11 Apo stereomicroscope at 6–66× magnification, provided with a drawing device. Electron micrographs (SEM) were obtained under a Zeiss EVOL LS10 scanning electron microscope at an accelerating voltage of 10 kV; ten seeds were directly mounted onto aluminum stubs with double adhesive tape and coated with gold prior to observation. The seed surface sculpturing terminology mainly followed Barthlott (1981, 1984) and Gontcharova et al. (2009). The vouchers are deposited in the Herbarium of the University of Catania (CAT). The herbarium codes are according to Thiers (2020). Phytosociological investigations were carried out using the method of the Sigmatist school (Braun-Blanquet 1928), while for the syntaxonomical arrangement, Mucina et al. (2016) was followed. The conservation status of the species was calculated with GeoCAT (Geospatial Conservation Assessment Tool) software (Bachman et al. 2011) and according to IUCN guidelines (IUCN 2022).

## ﻿Taxonomic treatment

### ﻿1. Solenopsis bivonae

Taxon classificationPlantaeAsteralesCampanulaceae

(Tineo) M.B.Crespo, Serra & A.Juan, Pl. Syst. Evol. 210(3–4): 219. 1998.

35F4EA10-2CA7-5EB6-BFA8-C5152695ED8B

≡ Lobeliabivonae Tineo, Cat. Pl. Hort. Reg. Panorm.: 279, 1827.
≡ Laurentiabivonae (Tineo) Pignatti, Giorn. Bot. Ital. 111:54, 1977.
≡ Lobeliatenella Biv., Sic. Pl. Cent. I: 53. 1806, non L., Mantissa Alt.: 120, 1771.
≡ Laurentiatenella A. DC., Prodr. 7(2): 410, 1839, p.p.
≡ Solenopsislaurentiasubsp.tenella (A. DC.) O. Bolòs et al., Fl. Manual Paisos Catalans: 1215. 1990, p.p.
≡ Laurentiagasparrinii(Tineo)Stroblsubsp.tenella (A. DC.) O. Bolòs & Vigo, Collect. Bot. (Barcelona) 14:102, 1983, p.p.
≡ Solenopsisbivonaeana C. Presl, Prodr. Mon. Lobel.: 32. 1836, p.p.

#### Type.

*Lobelia* 33* *tenella* Bivona, Cent. 1. p. 53. n. 58. Ad margines fluminis Oreti, *Bivona Bernardi* (lectotype: BM, designated by Crespo et al. 1998).

#### Description.

Perennial herb, acaulescent, rosulate, with 2–12.5 cm in diameter, provided with fibrose slender roots. Leaves 10–100 mm long, oblanceolate to spathulate, with blade entire or weakly crenate, glabrous, 4–40 × 2–15 mm, with petiole 3–60 mm long. Floral pedicels 2–11 cm, 2–3 times longer than leaves, with 1–2 bracteoles, 1.8–5.5 mm long, 0.1–0.7 mm wide, with glands at the margin. Calyx 3–5 mm long, with linear–lanceolate lobes, 2–4 mm long. Corolla 8.5–14.5 mm long, bilabiate, with tube 3.5–5 mm long, 0.9–1.5 mm in diameter; upper lip with two lobes linear–lanceolate, 3–6 mm long, 1.2–2.4 mm wide, bluish–lilac to dark lilac; lower lip trilobed, 5–9 mm long, widely edged in bluish–lilac and irregularly white in the central part until the base, covered by papillae in the ventral face. Stamen filaments free, 4–5.5 mm long, anthers violet, connate into a tube 1.4–1.9 mm long, wholly encapsulating the stigma; the two lower anthers are smaller, each appendiculate at the top with a tuft of hairs, closing a narrow fissure; the three upper anthers are curved. Ovary fused with the calyx tube; style whitish, 4–7 mm long; stigma pale lilac, bifid, papillate, with a ring of hairs just under the base. Capsule 1.6–3 mm long. Seeds more or less ellipsoid, shining, 0.40–0.50 × 0.2–0.3 mm.

### ﻿1.1. Solenopsis bivonae

Taxon classificationPlantaeAsteralesCampanulaceae

(Tineo) M.B.Crespo, Serra & A.Juan, Pl. Syst. Evol. 210(3–4): 219. 1998. subsp. bivonae

5992175A-92B7-50B4-830B-F351AFD46C19

**Figure 1.**
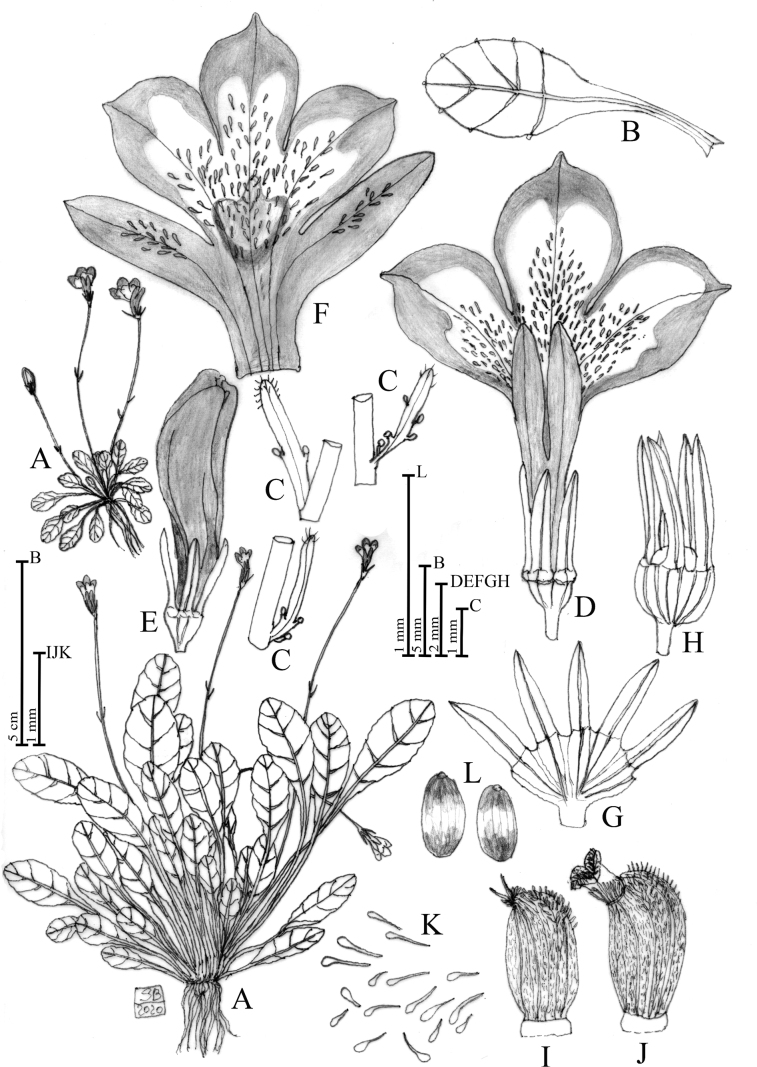
Solenopsisbivonaesubsp.bivonae**A** habit **B** leaf **C** bracts **D** flower in dorsal view **E** bud **F** open corolla **G** open calyx **H** calyx and capsule **I** anther in lateral view **J** anther in lateral view with exerted stigma **K** unicellular papillae occurring in the ventral face of the corolla **L** seeds. Drawn by Salvatore Brullo.

Figs 1 , 6A , 7A , 8C , 9A

#### Description.

Basal rosette 2–12.5 cm in diameter, with leaves 12–100 mm long, spathulate, with blade 6–40 × 4–15 mm and petiole 5–60 mm long; floral pedicels 5–11 cm, with (1) 2 bracteoles, very close near the middle, 2–2.4 mm long, 0.3–0.5 mm wide, hairy at the apex, with 1–4 stipulated glands at the margin per side; calyx 3–4 mm long, with lobes 2–3 mm long; corolla 10–12 mm long, with tube lilac, 4–5 mm long, ca.1 mm in diameter; upper lip with lobes 3.5–4.5 mm long, 1.3–1.7 mm wide, bluish–lilac, acute at apex, provided in the ventral face with papillae in the central part, 0.25–0.6 mm long; lower lip 5–7 mm long, with a small greenish–yellow macula at the base, slightly bordered of brown at base, lobes ovate and mucronate at the apex, 3.5–4.5 × 3–4 mm, covered by not very dense papillae for more than the lower half; stamen filaments 4–4.5 mm long, anther connate into a tube 1.5–1.8 mm long; the two lower anthers are without papillae at basis; the three upper anthers with hairs in the upper part of the back; style 4–4.5 mm long; capsule smooth, 1.6–2 mm long; seeds ellipsoid–fusiform, brownish, 0.40–0.45 × 0.2–0.25 mm.

#### Iconography.

Bivona-Bernardi (1806) tav. 2, sub *Lobeliatenella*; Boccone (1697) tav. 27, fig. top right, sub *Rapunculus aquaticus*, *minimus*, *repens*, *alpinus*, *bellidis folio*, *flore caeruleo inaperto*; Brullo et al. (2023b) Figs 2C, D, 4.

**Figure 2.**
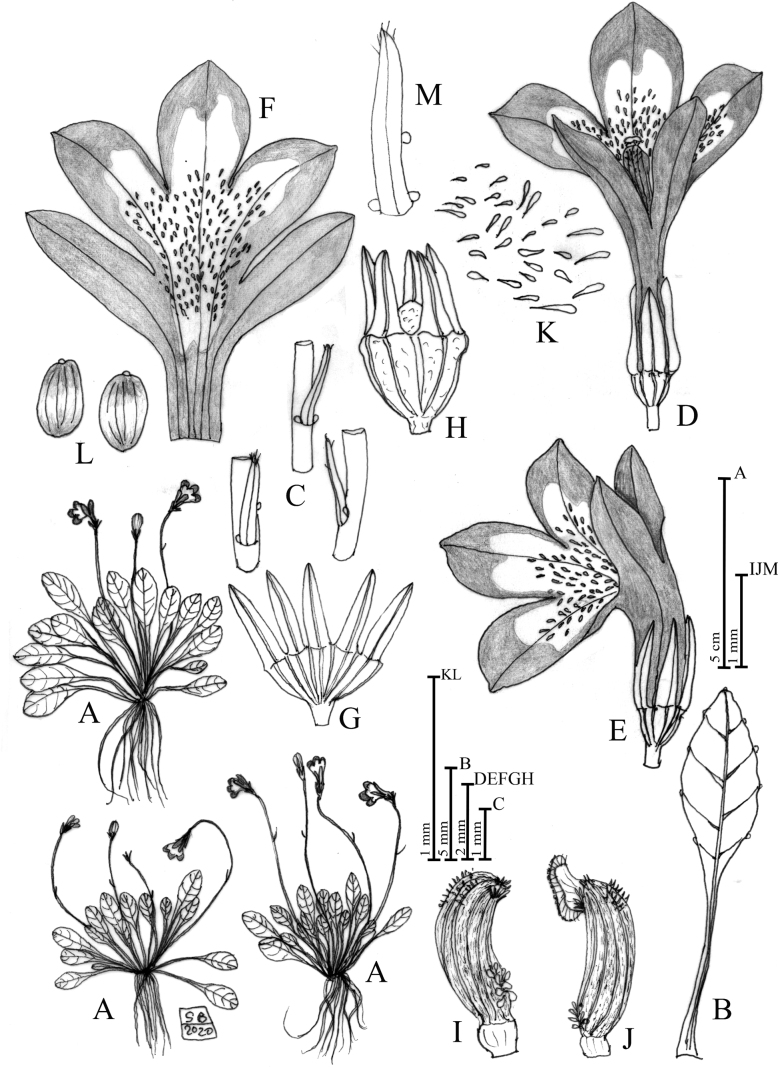
Solenopsisbivonaesubsp.madoniarum**A** habit **B** leaf **C** bracts **D** flower in dorsal view **E** flower in lateral view **F** open corolla **G** open calyx **H** calyx and capsule **I** anther in lateral view **J** anther in lateral view with exerted stigma **K** unicellular papillae occurring in the ventral face of the corolla **L** seeds **M** bract detail. Drawn by Salvatore Brullo.

#### Etymology.

It is dedicated to Antonino Bivona Bernardi, Sicilian botanist (1770–1837), who first described this species.

#### Phenology.

Flowering late April to September, fruiting May to September.

#### Distribution and ecology.

According to herbarium investigations and our field survey, this taxon occurs in North–West Sicily (Fig. 10), in the surroundings of Palermo, especially along the banks of the Oreto River, where it is today very rare, while it is very widespread along the Sosio river near Chiusa Sclafani. Previously, it was reported from Alcamo, where unfortunately it is extinct. As concerns its ecological requirements, it is localized at an elevation between 10 and 250 m, growing on calcareous vertical wet rocky places affected by permanent dripping (Fig. 8A). The plant community characterized by this hygrophyte can be referred to the phytosociological class *Adiantatea capilli–veneris* Br.-Bl. in Br.–Bl., Roussine and Nègre 1952 (cfr. Cambria 2020). This habitat is floristically differentiated by a moss carpet where grow also *Adiantum capillus–veneris* L. and *Samolusvalerandi* L.

#### Conservation status.

Currently, this taxon’s result is circumscribed in Sicily to two wet stands (Oreto and Sosio rivers), where it is very rare in the first locality and quite spread in the second one. Overall, this plant results in it being seriously threatened since it is linked to wetlands potentially subject to anthropic pressure, which tends to alter the water regime, prejudicing its survival. Therefore, in agreement with Conti et al. (1997), who quoted it as S.minutasubsp.nobilis, it can be treated as Endangered (EN), following IUCN criteria (IUCN 2022).

#### Additional specimens examined.

**Italy**, **Sicily**. Palermo in herbosis uliginosis, August 1888, *H. Ross s.n.* (PAL–GREUTER 8699, AMD43927); Sicile, 1831, *M. Tineo s.n.* (P00260397); Mondello, In locis hyeme inundatis, 1847, *M. Alb. de Franqueville s.n.* (P00260381); Orethus fluvius, locus rivulos, 1846, *M. Alb. de Franqueville s.n.* (P00260380); Palermo, s.d., *Tineo s.n.* (FI); Fiume Oreto, s.d., *Tineo s.n.* (FI); Fiume Oreto presso Palermo, s.d., *Parlatore s.n.* (FI); Palermo ad ripas F. Oreto, 25 April 1888, *N. Guzzino 3068* (AMD43928); Palermo: fiume Oreto, 13 May 1888, *D. Lanza s.n.* (AMD43930); Palermo ad fluviorum margines, May, *A. Todaro s.n.* (L2993294, FI, RO); Palermo, ad acquae dulcis in herbosis uliginosis, June 1895, *H. Ross 42* (L2993297, O-V2262582, FI); ex Sicilia, s. d., *G. Gussone s.n.* (L2993298); Palermo in herbosis uliginosis, June 1888, *H. Ross s.n.* (L2993299, RO); Palermo, ad rivulorum margines, August, *A. Todaro 463* (U1178908, P03406807, FI, RO); Fiume Oreto in humidis marginis, s.d., *A. Todaro s.n.* (U1178909); Palermo al fiume Oreto (Sicilia), in maritimis ad muros humidos, July 1881, *M. Lojacono s.n.* (P04608258, P00260396, MPU255098, FI); Ad muros madidos, Palermo, July, *M. Lojacono s.n.* (PAL39476); Palermo, June 1889, *A. Todaro s.n.* (P00260403); In humidis ad muros prope Panormum, 20 May 1855, *E. & A. Huet du Pavillon* (O-V2263343, FI); Lungo l’Oreto a Palermo, 22 August 1902, *A. Mazza s.n.* (FI); Panormi, ad rivulos alla Guadagna, September 1869, *F. Parlatore s.n.* (FI); Palermo a S. Maria di Gesù, in luoghi umidi, 1 May 1895, *Biondi s.n.* (FI); Fiume Oreto presso la Guadagna e S. Erasmo, June 1834, *F. Parlatore s.n.* (FI); Palermo alla Guadagna, 29 September 1868, *F. Parlatore s.n.* (FI); In humidis Palermo, s.d., *A. Todaro s.n.* (RO); Fiume Oreto, 1817, *Tineo s.n.* (RO); Fiume Oreto, Palermo, 38°5'18.17"N, 13°20'35.45"E, 46 m, 29 July 2018, *S. Cambria s.n.* (CAT); Alcamo in humentibus arenosis, May, *Citarda 241* (RO); Fiume Sosio, S. Carlo, Chiusa Sclafani, 19 August 1995, *G. Certa s.n.* (PAL89386); Contrada Tagliarini près du fleuve Sosio, commune de Prizzi, province de Palerme, Sicile, Altitude: m. 640 environ. Le long bords humides. 20 August 1996, *G. Certa s.n.* (PAL39475); Fiume Sosio, 28 August 1986, *G. Spampinato s.n.* (CAT037289); Fiume Sosio, località S. Carlo (Chiusa Sclafani), 37°38'22.13"N, 13°15'59.66"E, 223 m, 10 July 2018, *S. Cambria & G. Di Gregorio s.n.* (CAT).

### ﻿1.2. Solenopsis bivonae

Taxon classificationPlantaeAsteralesCampanulaceae

(Tineo) M.B.Crespo, Serra & A.Juan subsp. madoniarum Brullo, C. Brullo, Cambria, Tomaselli, Minissale & Giusso del Galdo subsp. nov.

A95CAD74-CC4B-587E-94CB-B289829A5FF1

urn:lsid:ipni.org:names:77323164-1

Figs 2 , 6D , 7B , 8D , 9B

#### Type.

Italy. Sicily. Madonie, laghetto sopra Piano Zucchi, 37°52'43.40"N, 14°0'12.87"E, 1259 m a.s.l., 15 July 2018, *S. Cambria s.n.* (holotype CAT).

#### Diagnosis.

It differs from the type in having leaves arranged in a smaller rosette with shorter blade, shorter floral pedicel, provided with a single bracteole glabrous or with few apical hairs and 1–2 basal sessile glands, corolla smaller with upper lip lobes without glands in the ventral face and lower lip lobes shorter, obtuse, provided with dense and shorter papillae, with anther tube papillose at the basis and longer capsule. Conversely, the type is characterized by leaves arranged in a larger rosette with longer blade, longer floral pedicel, provided with (1)2 bracteoles with several hairs at the apex and 1–4 lateral stipulated glands, corolla larger with upper lip lobes with glands in the ventral face and lower lip lobes longer, acute, provided with lax and longer papillae, anther tube without papillae at the basis and shorter capsule.

#### Description.

Basal rosette 3.5–8 cm in diameter, with leaves 15–45 mm long, oblanceolate to oblanceolate–spathulate, with blade 4–20 × 2–8 mm and petiole 8–25 mm long; floral pedicels 2–5(9) cm, with one bracteole near the middle, 1.8–2.2 mm long, 0.1–0.3 mm wide, with few hairs at the apex, with 1 or 2 sessile glands at the base and rarely one sessile gland at the margin; calyx 3–4 mm long, with lobes 2–3.5 mm long; corolla 8.5–10 mm long, with tube lilac, 3.7–4.5 mm long, 0.9–1.3 mm in diameter; upper lip with lobes 3–4 mm long, 1.2–1.7 mm wide, bluish–lilac, obtuse or slightly mucronate at apex, provided in the ventral face with dense papillae in the lower half, 0.1–0.24 mm long; lower lip 5–6 mm long, with a large yellowish macula at the base, bordered at the base by a brown band, lobes ovate and obtuse or slightly mucronate at the apex, 2.5–3.5 × 1.6–2.5 mm, covered by dense papillae in the lower half; anther connate into a tube 1.4–1.6 mm long; the two lower anthers are papillose at the base; style 4.5–5.5 mm long; capsule 2.7–3 mm long; seeds obovoid-ellipsoid, pale brown, 0.40–0.46 × 0.24–0.26 mm.

#### Etymology.

The epithet derives from Madonie, a massif of North Sicily, where this taxon is rather spread.

#### Phenology.

Flowering late May to October, fruiting June to October.

#### Distribution and ecology.

Based on herbarium data and field investigations, this taxon is distributed in the Madonie massif, where it is localized in many places at 700-1600 m of altitude (Fig. 10), represented mainly by peat bogs, dominated by *Sphagnum* sp. pl., *Aulacomniumpalustre* (Hedw.) Schwägr. and *Polytrichumcommune* Hedw. Here, it characterized an orophilous plant community belonging to *Scheuchzerio palustris–Caricetea fuscae* R. Tx. 1937, as emphasized by Raimondo et al. (1980, 2021). Sometimes, as near Petralia Soprana or Piazza Armerina, it occurs also on calcareous vertical wet rocky places affected by permanent dripping, where it is a member of vegetation of the class *Adiantetea capilli–veneris*, dominated by *Adiantum capillus–veneris* and several bryophytes.

#### Conservation status.

This taxon shows a scattered distribution, occurring mainly in some localities within the Madonie Regional Park. Besides, it is a species closely linked to small wet stands fed by water springs, whose collecting leads to the destruction of the habitat and the disappearance of the vegetation that characterizes it. It shows an EOO of 410 km^2^ and an AOO of 20 Km^2^. Therefore, according to B criterion, we propose to consider this taxon as Endangered [EN – B1ab(iii)+2ab(iii)) (IUCN 2022)].

#### Additional specimens examined

**(paratypes). Italy, Sicily.** Madonië, van Portella Mandarini naar Geraci Siculi, op bult in moeras, c. 1400 m., 9 June 1983, *J. Mennema 2962* (L2993484); Ad rivulos et fontes Montium Nebrodensium (alla fontana di S. Nicolò sul M. Pietrafucile, 24 June 1840, *De Heldreich s.n.* (P00260388; WAG1507801, FI); Italie, Sicile, Prov. Palermo, entre Portella Mandarini (1206 m) et Geraci (1070 m) en passant pour la base de Punta Argentiera (1450 m), 9 June 1983, *A. Charpin, M. Dittrich & D. Jeanmonod 96449* (PAL); Ad aquas scaturientes Madoniarum 3500’, 6 August 1874, *G. Strobl s.n.* (FI); Ad scaturigines frigidas Nebrodes acque delle Favare di Petralia, July 1888, *M. Lojacono 319* (FI); Madonie presso il passo della Botte, July 1904, *F. Cavara s.n.* (FI); Madonie a Vulpignano, alla Favara, a Polizzi presso alla Pietà, June 1840, *F. Parlatore s.n.* (FI); A montibus nebrodensibus, s.d., *Schouw s.n.* (G-DC00239486); Contrada Scorzone (Geraci Siculo), 22 June 2004, *R. Galesi s.n.* (CAT000194); Piano Pomo (Madonie-PA), 31 July 1990, *Bartolo, Brullo, Pulvirenti, Scelsi, Spampinato s.n.* (CAT037288); Madonie, Portella Mandarini, sfagnete, 37°51'55"N, 14°07'04"E, 1247 m, 15 July 2018, *S. Cambria s.n.* (CAT); Madonie, Petralia Soprana, sorgente Cataratta, parete umida, 37°49'37.26"N, 14°4'11.39"E, 1166 m, 15 July 2017, S. *Cambria s.n.* (CAT); Piazza Armerina, Monte Canalotto, presso l’abbeveratoio, 37°28'6.55"N, 14°22'41.04"E, 771 m, 16 October 2021, *S. Cambria & D. Azzaro s.n.* (CAT).

### ﻿1.3. Solenopsis bivonae

Taxon classificationPlantaeAsteralesCampanulaceae

(Tineo) M.B.Crespo, Serra & A.Juan subsp. peloritana Brullo, C.Brullo, Cambria, Tomaselli, Crisafulli, Minissale & Giusso del Galdo subsp. nov.

5F2D047E-5942-57B5-A80D-AC9DABECCD6B

urn:lsid:ipni.org:names:77323165-1

**Figure 3.**
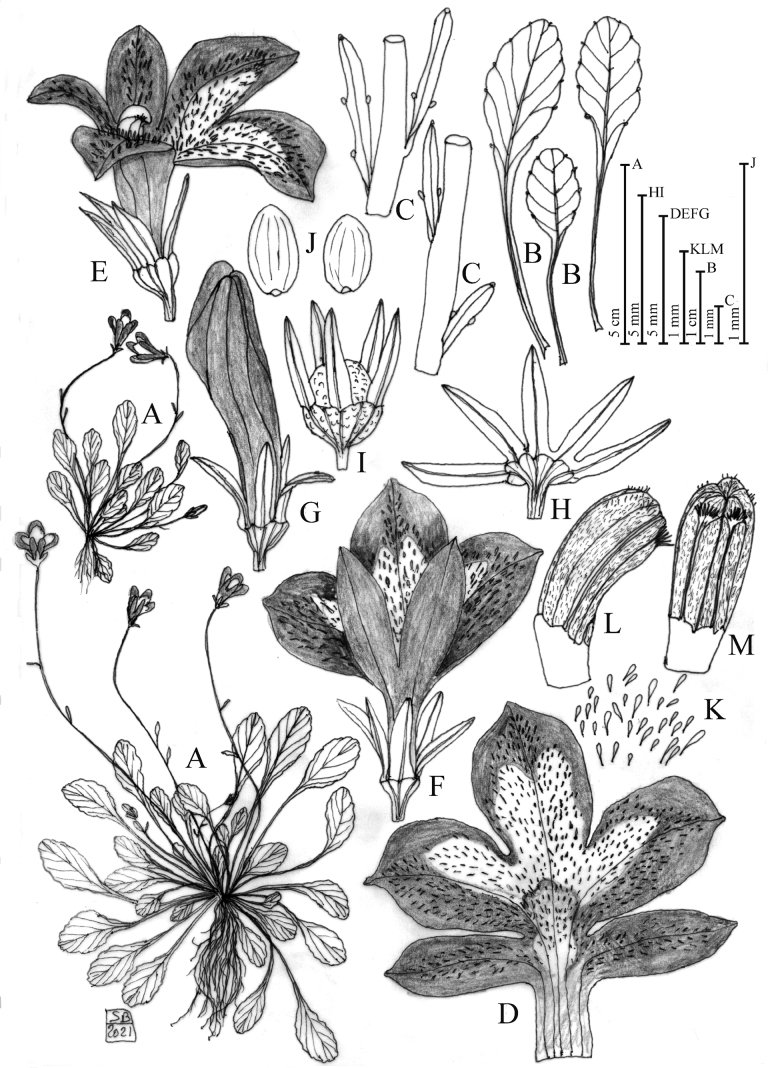
Solenopsisbivonaesubsp.peloritana**A** habit **B** leaves **C** bracts **D** open corolla **E** flower in lateral view **F** flower in dorsal view **G** bud. **H** open calyx **I** calyx and capsule **J** seeds **K** unicellular papillae occurring in the ventral face of the corolla **L** anther in lateral view **M** anther in ventral view. Drawn by Salvatore Brullo.

Figs 3 , 6B , 7C , 8E , 9C

#### Type.

Italy. Sicily. Monti Peloritani, Vallone Passo Pirtuso, S. Lucia del Mela, 38°4'59"N, 15°18'28"E, 559 m, 19 July 2020, *S. Cambria, A. Crisafulli & F. Anania s.n.* (holotype CAT).

#### Diagnosis.

It differs from the type in having longer bracteoles, glabrous, provided with apical gland, longer calyx with longer lobes, larger corolla with denser and spread glands in the ventral face, larger upper lip lobes and lower lip lobes, within the lower lip a yellow macula at the base, slightly bordered of red–brown, longer style and larger capsule. Conversely, the type is characterized by shorter bracteoles, hairy apex without gland, shorter calyx with shorter lobes, smaller corolla with more scattered glands in the ventral face, smaller upper lip lobes and lower lip lobes, within the lower lip a greenish-yellow macula at the base, slightly bordered of brown, shorter style and smaller capsule.

#### Description.

Basal rosette 4–10 cm in diameter, with leaves 15–55 mm long, with blade 7–23 × 4–10 mm and petiole 5–30 mm long; floral pedicels 5.5–11 cm, with 2 bracteoles, 3–5.5 mm long, 0.4–0.7 mm wide, glabrous, with one terminal gland and 1–2 stipulated glands at the margin per side; calyx 4–5 mm long, with lobes 3.2–4 mm long; corolla 12–14.5 mm long, with tube green with lilac ribs, 3.5–4 mm long, ca. 1.5 mm in diameter; upper lip with lobes 5–6 mm long, 2–2.4 mm wide, dark lilac, provided in the ventral face with papillae in the central part, 0.1–0.4 mm long; lower lip 8–9 mm long, with a small yellow macula at the base, slightly bordered of red-brown at base in the upper part or sometimes with central red–brown spot, lobes obovate, the central one 5.5–6.5 × 4–4.5 mm, the lateral ones 4.5–5.5 × 4–4.2 covered by very dense papillae almost until the apex; stamen filaments 4.5–4.7 mm long, anther connate into a tube 1.7–1.9 mm long; the three upper anthers with scattered hairs in the upper part of the back; style 6.5–7 mm long; capsule smooth, 2.5–3 mm long; seeds ellipsoid, 0.45–0.50 × 0.24–0.26 mm.

#### Etymology.

The epithet derives from Peloritani, a chain of North–eastern Sicily, where this taxon is localized.

#### Phenology.

Flowering June to August, fruiting July to August.

#### Distribution and ecology.

It grows on metamorphic vertical wet rocky stands affected by permanent dripping. It is a member of a plant community of the class *Adiantetea capilli–veneris*, dominated by *Adiantum capillus–veneris*, associated with *Samolusvalerandi* L., *Lysimachianemorum* L., HypericumhircinumL.subsp.majus (Aiton) N. Robson and several bryophytes. In this stand, it is localized exclusively along a short watercourse of the Mela valley (Peloritani chain) at an elevation of 600–700 m (Fig. 10), where several individuals of this taxon were surveyed.

#### Conservation status.

This taxon is known for one stand of the Peloritani chain, along a short wet wall, where about one hundred well-developed individuals were observed. This population is very isolated and inaccessible and it does not seem subject to immediate threats. It shows an EOO of 4 km^2^ and an AOO of 4 Km^2^. Therefore, according to the B criterion (IUCN 2022), we propose to consider this taxon as Critically Endangered category [(CR – B1ab(iii)+2ab(iii))].

### ﻿1.4 Solenopsis bivonae

Taxon classificationPlantaeAsteralesCampanulaceae

(Tineo) M.B.Crespo, Serra & A.Juan subsp. brutia Brullo, C.Brullo, Cambria, Tomaselli, Minissale & Giusso subsp. nov.

94C92AE2-DBEA-5F22-9277-F08661BE80EE

urn:lsid:ipni.org:names:77323166-1

**Figure 4.**
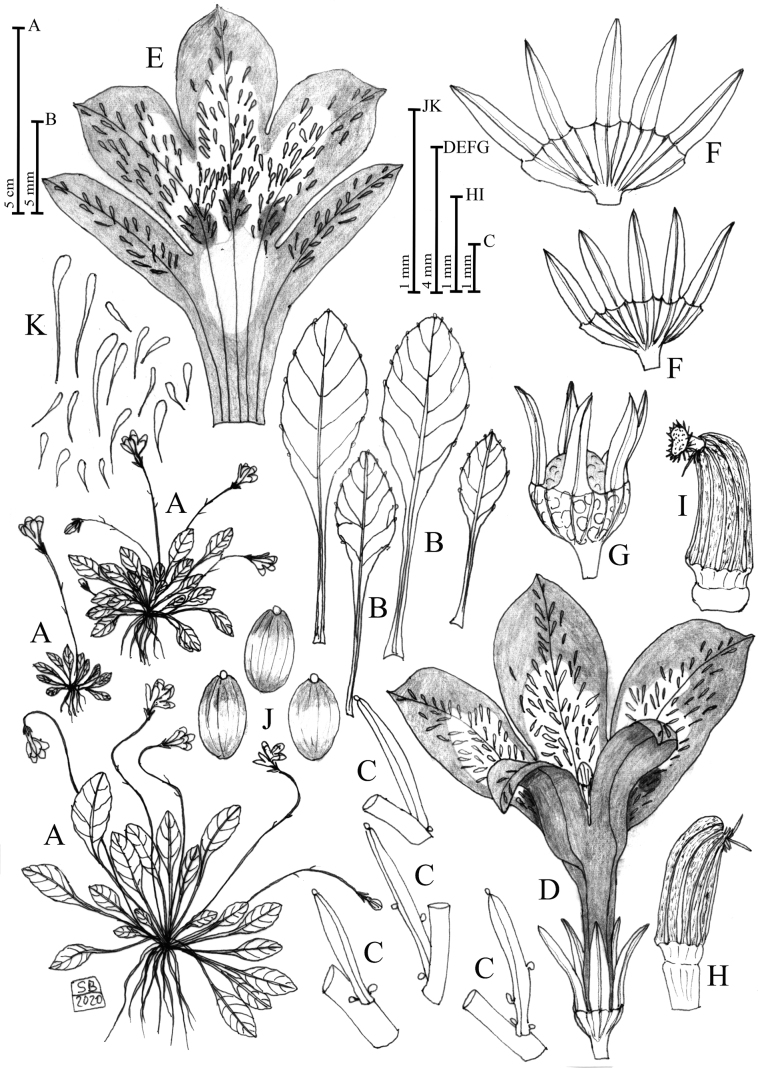
Solenopsisbivonaesubsp.brutia**A** habit **B** leaves **C** bracts **D** flower in dorsal view **E** open corolla **F** open calyces **G** calyx and capsule **H** anther in lateral view **I** anther in lateral view with stigma. **J** seeds **K** unicellular papillae occurring in the ventral face of the corolla. Drawn by Salvatore Brullo.

Figs 4 , 6C , 7D , 8F , 9D

#### Type.

Italy. Calabria. Rive del fiume Lao, presso Papasidero (Cosenza), 39°52'10.96"N, 15°54'7.93"E, 130 m, 06 August 2018, *S. Brullo, D. Puntillo & D. Uzunov s.n.* (holotype CAT).

#### Diagnosis.

It differs from the type in having leaves arranged in a smaller rosette, shorter leaves, with oblanceolate to oblanceolate-spathulate blade, shorter petiole, shorter floral pedicel, glabrous bracteoles, located in the upper half, provided with sessile apical gland, two basal glands and 0–2 lateral glands, corolla in the lower lip with a green macula at the base and provided with three dark blue spots above, lobes papillose up to the apex, longer staminal filaments, glabrous anther tube, longer style, slightly tuberculate capsule, reddish–brown and larger seeds. Conversely, the type is characterized by leaves arranged in a larger rosette, longer leaves, with spathulate blade, longer petiole, longer floral pedicel, bracteoles hairy at the apex, located in the middle, provided with 1–4 stipulated lateral glands, corolla in the lower lip with a greenish-yellow macula at the base, without spots, lobes papillose for more than the lower half, shorter staminal filaments, anther tube hairy at the apex, shorter style, smooth capsule, brownish and smaller seeds.

#### Description.

Basal rosette 2.5–7 cm in diameter, with leaves 10–58 mm long, oblanceolate to oblanceolate–spathulate, with blade 5–22 × 2–10 mm and petiole 3–36 mm long; floral pedicels 3–6 cm, with 2 bracteoles, spaced in the upper half, 2–3 mm long, 0.25–0.35 mm wide, glabrous with a sessile gland at the apex, with 2 basal sessile glands and 0–2 sessile glands at the margin; calyx 3.5–5 mm long; corolla 11–12 mm long, with tube white-lilac, 4.5–5 mm long, 1–1.2 mm in diameter; upper lip with lobes 4–4.5 mm long, 1.4–1.8 mm wide, sub–obtuse at apex, provided in the ventral face with papillae in the central part, 0.16–0.6 mm long; lower lip 6.5–7 mm long, greenish at the throat, surmounted by three distinct dark blue spots, slightly bordered of brown at base, lobes 3.5–5 × 2.5–3.5 mm, covered by not very dense papillae often almost to the apex; stamen filaments 5–5.5 mm long, anther connate into a tube 1.5–1.6 mm long; the three upper anthers glabrous in the upper part of the back; style 6–6.5 mm long; capsule slightly tubercolate, 2.3–3 mm long; seeds ellipsoid, reddish–brownish, 0.46–0.50 × 0.26–0.3 mm.

#### Phenology.

Flowering June to September, fruiting June to September.

#### Etymology.

The specific epithet refers to “Brutia,” the Latin name of Calabria, territory where this taxon grows.

#### Distribution and ecology.

This taxon was surveyed in the lower reaches of Lao river (North Calabria), at elevations of 130–350 m, where it grows on rocky metamorphic outcrops (Fig. 10). It likes humid and shady stands covered by a dense moss carpet, associated mainly to *Adiantum capillus–veneris*. As for the other subspecies previously examined, it is linked to hygrophilous communities of the *Adiantetea capilli–veneris* too. From a phytogeographical point of view, it should be noted that this taxon is the only *Solenopsis* with a perennial habit, localized in a continental territory since all the others occur exclusively in big Mediterranean islands (Crespo et al. 1998).

#### Conservation status.

The populations of this subspecies are rare and all circumscribed to the banks of Lao river in North–West Calabria. Based on recent field surveys, its presence in the three hitherto known locations has been confirmed in only one of them (near Papasidero), while in the other two, it seems to have disappeared (Laino–Castello and Laino–Borgo). It shows an EOO of 9.51 km^2^ and an AOO of 12 Km^2^. Therefore, in addition to its rarity and the considerable reduction of its current range, according to B criterion (IUCN 2022), we propose to consider this taxon as Endangered [EN – B1ab(iii)+2ab(iii)].

#### Additional specimens examined

**(paratypes). Italy, Calabria.** Valle del Lao (sopra le rocce e in altri luoghi umidi lungo il f. Lao ai piedi lo Borgo-Laino-Castello), 18 August 1892, *B. Longo s.n.* (RO); Sulle rocce umide lungo il fiume Lao alla Maradosa (Laino Castello), 27 September 1900, *B. Longo s.n.* (RO); Sopra una roccia umida lungo il fiume Lao (Laino Castello-Cosenza), 16 August 1902, *B. Longo s.n.* (RO).

### ﻿2. Solenopsis meikleana

Taxon classificationPlantaeAsteralesCampanulaceae

Brullo, C.Brullo, Cambria, Tomaselli, Minissale & Giusso, sp. nov.

48FADE9D-294F-5A8F-9AD9-C88F27B41C27

urn:lsid:ipni.org:names:77323167-1

**Figure 5.**
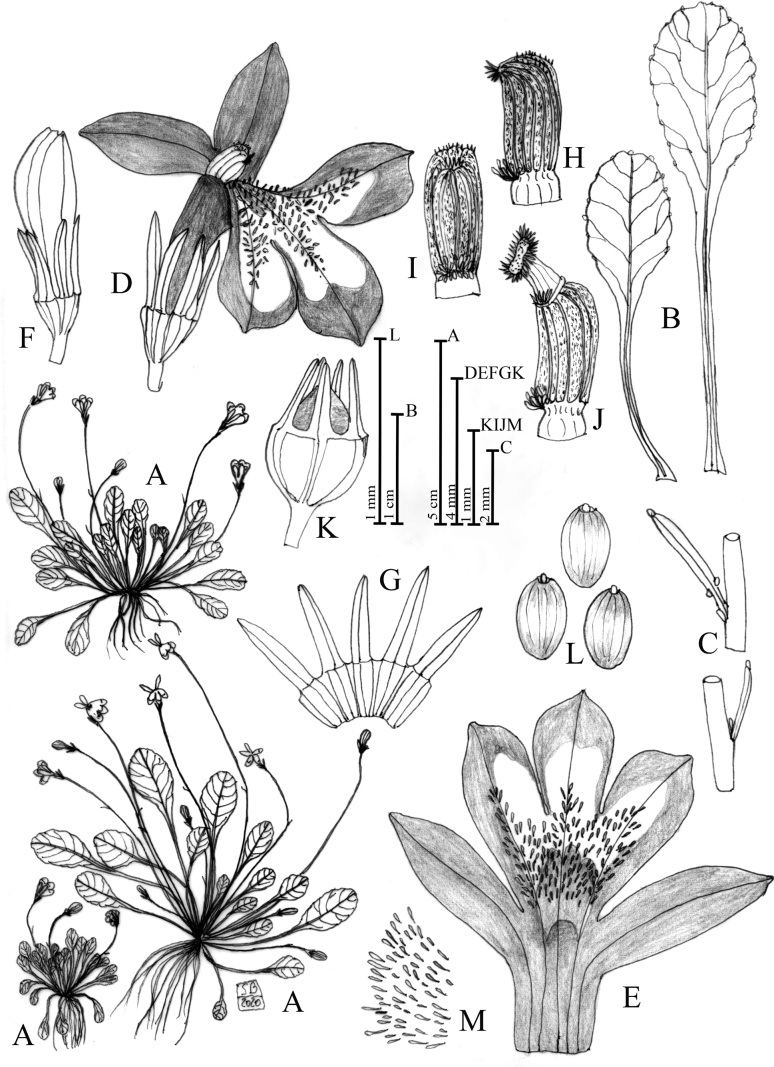
*Solenopsismeikleana* sp. nov. **A** habit **B** leaves **C** bracts **D** flower in lateral view **E** open corolla **F** bud **G** open calyx **H** anther in lateral view **I** anther in ventral view **J** anther in lateral view with stigma **K** calyx and capsule **L** seeds **M** unicellular papillae occurring in the ventral face of the corolla. Drawn by Salvatore Brullo.

**Figure 6.**
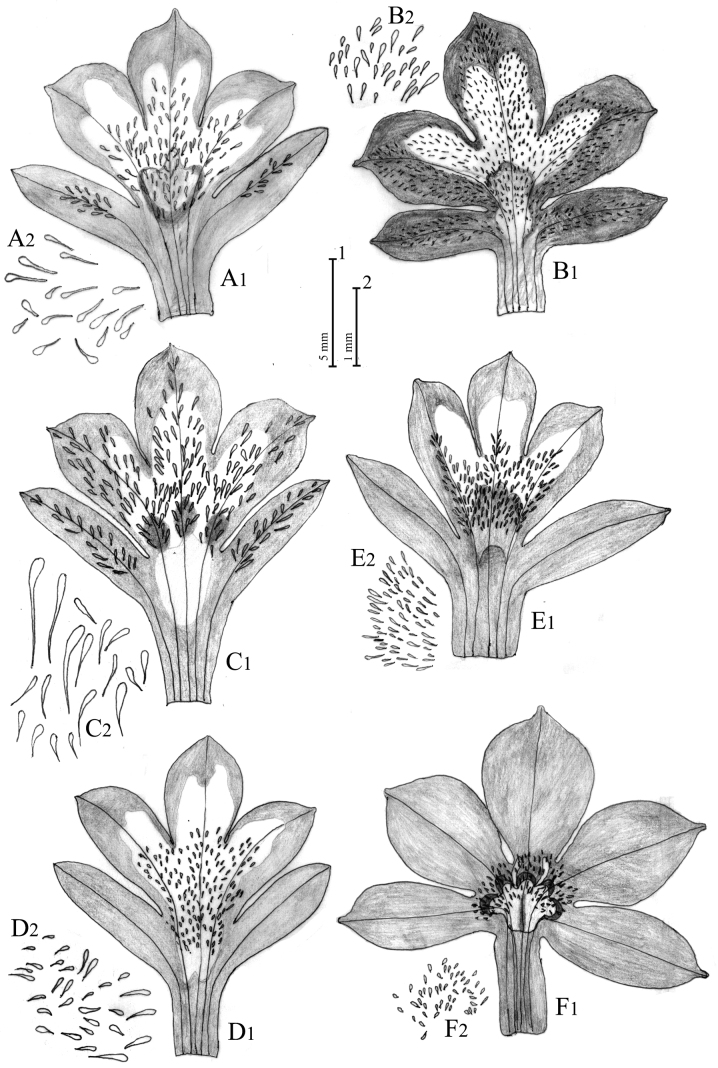
Open corolla (**1**) and detail of corolla papillae (**2**) of Solenopsisbivonaesubsp.bivonae (**A**), S.bivonaesubsp.peloritana (**B**), S.bivonaesubsp.brutia (**C**), S.bivonaesubsp.madoniarum (**D**), *S.meikleana* (**E**) and *S.bacchettae* (**F**). Drawn by Salvatore Brullo.

**Figure 7.**
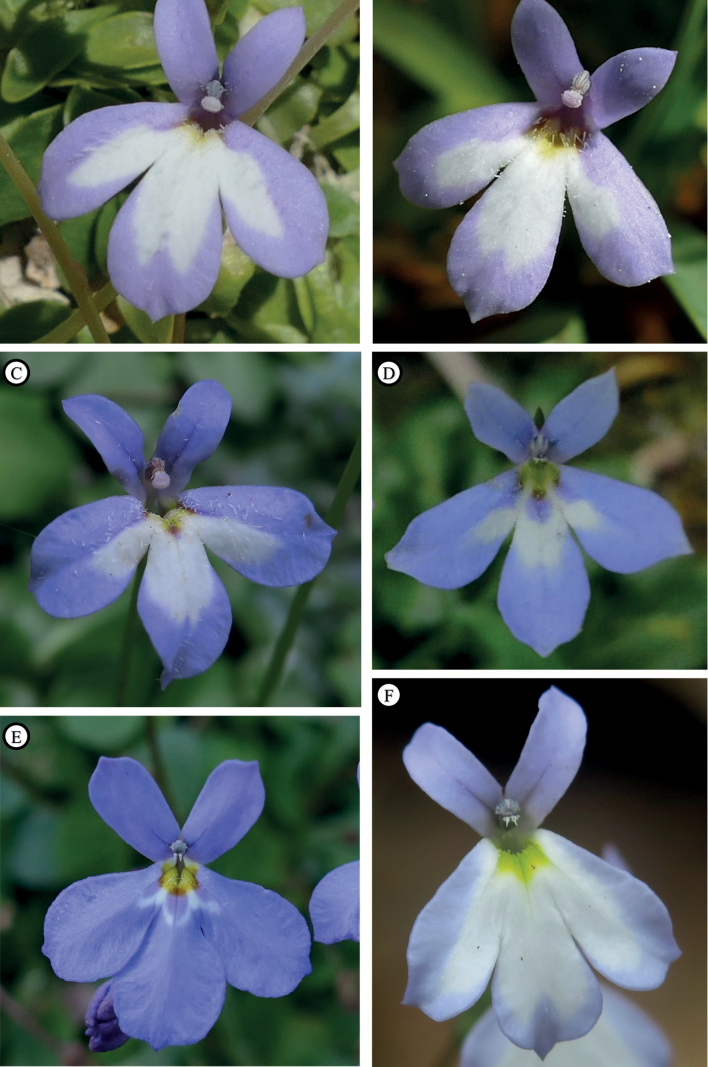
Flowers in frontal view in natural habitat of Solenopsisbivonaesubsp.bivonae (**A**), S.bivonaesubsp.madoniarum (**B**), S.bivonaesubsp.peloritana (**C**), S.bivonaesubsp.brutia (**D**), *S.bacchettae* (**E**) and *S.meikleana* (**F**). Photographed by Salvatore Cambria (**A–C, E, F**) and Lorenzo Peruzzi (**D**).

**Figure 8.**
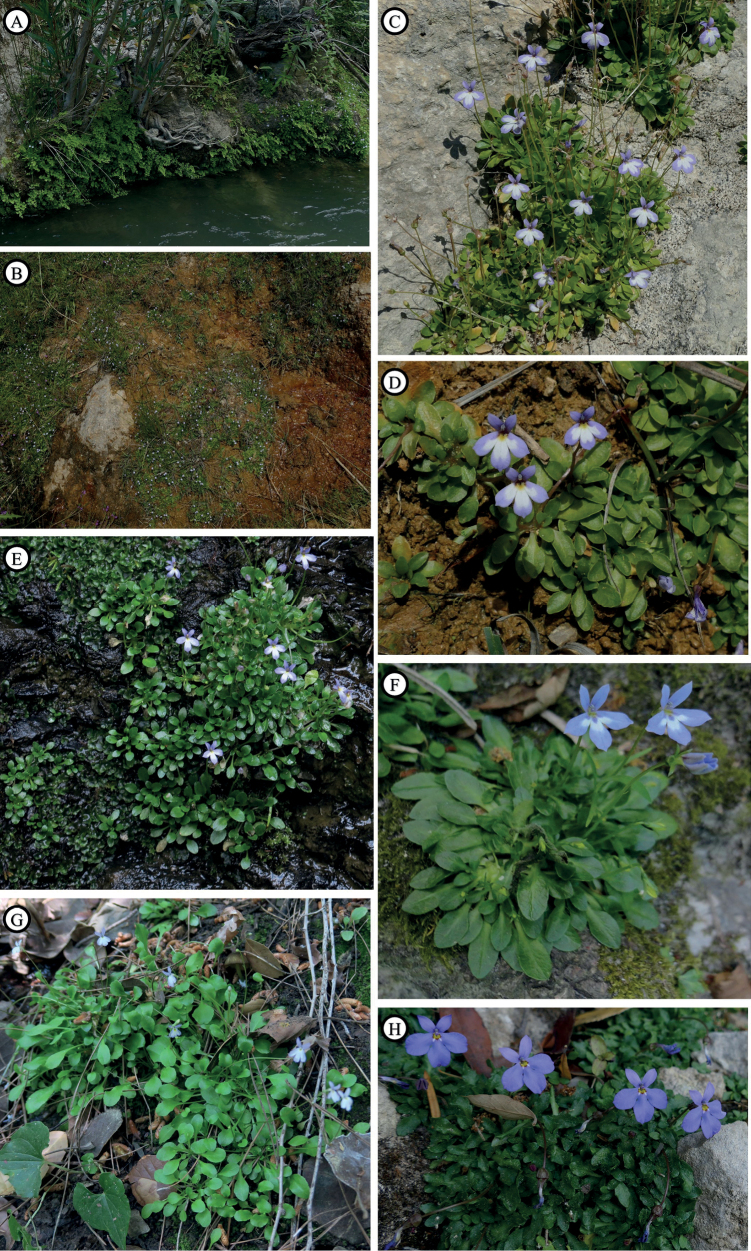
Natural habitat along the Sosio river (Sicily) colonized by Solenopsisbivonaesubsp.bivonae (**A**). Natural habitat in Madonie massif (Sicily) colonized by S.bivonaesubsp.madoniarum (**B**). Habit of S.bivonaesubsp.bivonae from Sosio River (**C**). Habit of S.bivonaesubsp.madoniarum from Madonie massif (**D**). Habit of S.bivonaesubsp.peloritana from Mela River, Sicily (**E**). Habit of S.bivonaesubsp.brutia from Lao River, Calabria (**F**). Habit of *S.meikleana* from Cedar Valley, Cyprus (**G**). Habit of *S.bacchettae* from Seui, Sardinia (**H**). Photographed by Salvatore Cambria (**A–E, G, H**) and Lorenzo Peruzzi (**F**).

**Figure 9.**
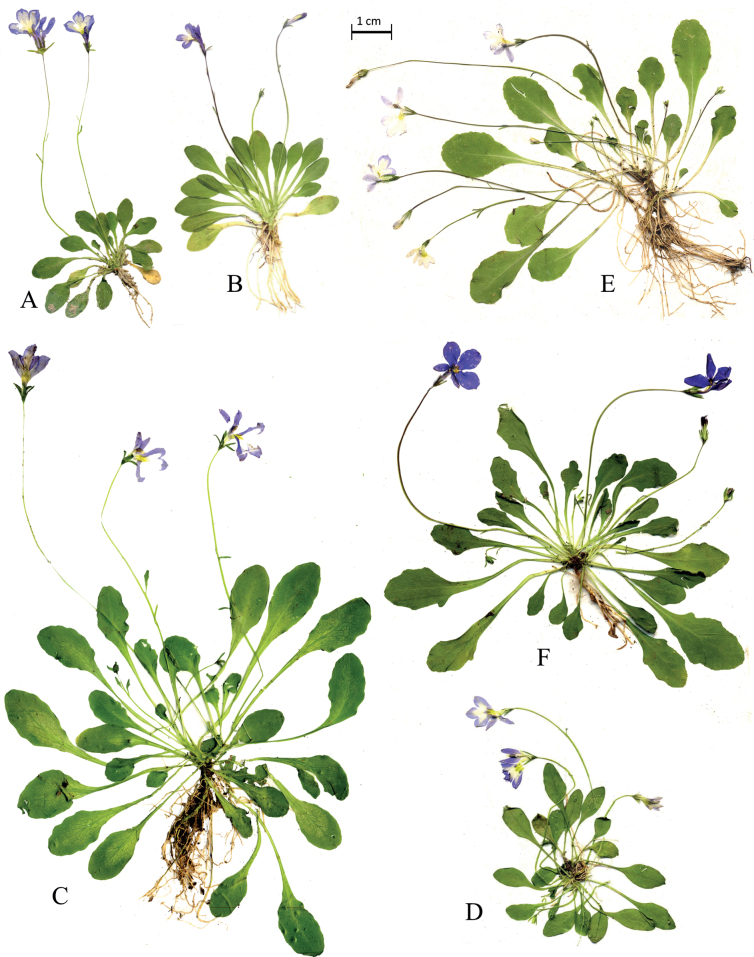
Habit of living plants of Solenopsisbivonaesubsp.bivonae from Sosio river (**A**), S.bivonaesubsp.madoniarum from Madonie massif (**B**). S.bivonaesubsp.peloritana from Mela River (**C**). Habit of S.bivonaesubsp.brutia from Lao River (**D**). *S.meikleana* from Cedar Valley, Cyprus (**E**). *S.bacchettae* from Seui, Sardinia (**F**).

Figs 5 , 6E , 7F , 8G , 9E

Laurentia tenella Auct. Fl. Cypr., non A. DC. Prodr. 7(2): 410, 1839.
Laurentia minuta Auct. Fl. Cypr., non A. DC. Prodr. 7(2): 410, 1839.
Laurentia minuta (L.) DC. f. nobilis Wimmer, Ann. Naturhist. Mus. Wien 56:333, 1948, p.p.
Solenopsis minuta (L.) C. Presl subsp. nobilis (Wimmer) Meikle, Kew Bull. 34(2): 374, 1979, p.p.
Solenopsis bivonae (Tineo) M. B. Crespo, Serra & A. Juan, Pl. Syst. Evol. 210(3–4): 219. 1998, p.p.
Solenopsis bivonae Christodoulou et al., Cypricola 17: 1, 2020, p.p.

#### Type.

Cyprus. Mesa Potamos Falls, 34°53'31.88"N, 32°54'32.37"E, 960 m, 6 June 2019, *S. Cambria s.n.* (holotype CAT).

#### Diagnosis.

It differs from *Solenopsisbivonae* in having glabrous and longer bracteoles, provided with apical sessile glands and 1––2 glands per side, pale blue or pale violet corolla, with upper lip lobes without papillae, lower lip lobes oblong, smaller, provided with shorter glands, anther tube shorter and papillose at the base, shorter style and longer capsule. Conversely, *S.bivonae* is characterized by shorter bracteoles, hairy at the apex and with 1––4 glands per side, bluish-lilac corolla, with upper lip lobes with papillae in the ventral face, lower lip lobes linear-lanceolate, larger, provided with longer glands, anther tube longer, without basal papillae, longer style and shorter capsule.

#### Description.

Basal rosette 2.5–11 cm in diameter, with leaves 10–75 mm long, oblanceolate–spathulate, with blade glabrous or covered by scattered hyaline hairs, 6–30 × 4–15 mm and petiole 5–50 mm long; floral pedicels 2–12 cm, subequal to 3 times longer than leaves, with 1–2 bracteoles, 2–8 mm long, 0.2–0.6 mm wide, glabrous, with 1–2 stipulated glands at the margin per side; calyx 3–5 mm long, with lobes 1.5–3 mm long; corolla 10–12 mm long, with tube green-violet, 3–5 mm long, 1.1–1.3 mm in diameter; upper lip with lobes 1.5–1.7 mm wide, pale blue to pale–violet, without papillae; lower lip 5–5.5 mm long, lobes oblong, obtuse at the apex, 2.5–3.5 × 1.4–2.2 mm, covered by papillae 0.05–0.3 mm long; stamen filaments 3–5 mm long, anther connate into a tube 1–1.5 mm long; the two lower anthers are papillose at basis; the three upper anthers with scattered hairs in the upper part of the back; style 3.5–4 mm long; capsule 3–3.2 mm long; seeds broadly ellipsoid, 0.40–0.46 × 0.24–0.29 mm.

#### Iconography.

Meikle (1985), plate 65.

#### Phenology.

Flowering March to October, fruiting April to October.

#### Etymology.

It is dedicated to Robert Desmond Meikle (1923–2021), author of the “Flora of Cyprus,” who dealt with the taxonomy of the genus *Solenopsis*.

#### Distribution and ecology.

**Figure 10.**
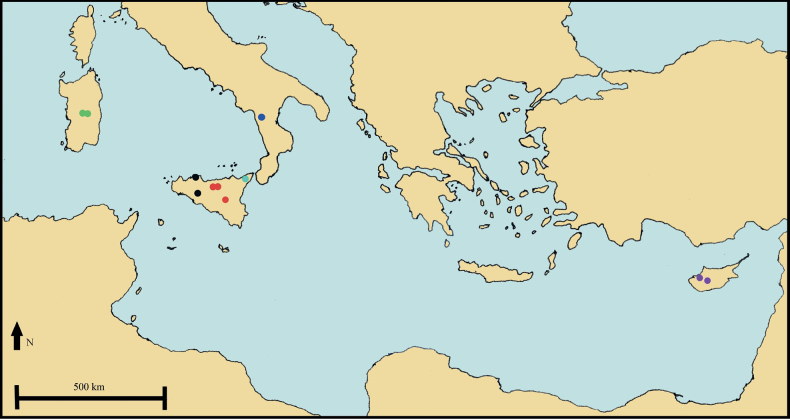
Geographical distribution of Solenopsisbivonaesubsp.bivonae (black dots); S.bivonaesubsp.madoniarum (red dots); S.bivonaesubsp.peloritana (pale blue dot); S.bivonaesubsp.brutia (dark blue dot); *S.meikleana* (purple dots); *S.bacchettae* (green dots).

This species occurs in western Cyprus, where it is localized in very moist environments such as river banks, springs, waterfalls, and dripping walls (Fig. 10). Usually, it grows from hills to mountain belts up to an elevation of 1600 m, on ophiolitic rocky outcrops covered by bryophyte carpets and ferns, particularly *Adiantum capillus–veneris*. This vegetation can be referred to the *Adiantetea capilli–veneris* class for its floristic and ecological peculiarity.

#### Conservation status.

This species, endemic to Cyprus, shows a scattered distribution in the western part of the island. It is a perennial hygrophyte, usually occurring in the wet rocky stands, which are always subject to dripping waters. Regarding conservation, the habitat characterized by this species is subject to synanthropic threats, represented mainly by the uptake of springs or the waters of streams, which allow its survival. The species shows an EOO of 1298 km^2^ and an AOO of 288 Km^2^. Therefore, according to B criterion (IUCN 2022), we propose to consider this taxon as Endangered [EN – B1ab(iii)+2ab(iii)].

#### Additional specimens examined

**(paratypes). Cyprus**. Iter Cyprium, Mont Troodos 5000–6400‘, 10 June 1912, *M. Haradjian s.n.* (L2993291); frequens ad fontes in pago Moni inter Larnaca et Limassol, 28 April 1862, *T. Kotschy 576* (L2993300, G-BOIS00781682); Troodos, valley Caledonian falls. On rocks next to the falls. 34°54'N, 32°52'E, Alt. 1350, 22 July 1995, *J.J. Wieringa 3330 and M.I.D. Janzen* (WAG 1335512); Iter Cyprium, pr. Galata, 16 June 1880, *Sintenis et Rigo 742* (P00260376); Ganze voicin de la Gratiola et de la Bonnaga in insula Cypri in humidis maritimis, 1837, *M. Aucher-Eloy s.n.* (P00260370); in Cypro, s.d., *M. Aucher-Eloy 3854* (P00260371, G-BOIS00781706); In humidis in insulae Cypri, 1836, *M. Aucher (Eloy) s.n.* (G-DC00329488); Ins. Cypro, in valle fluminis prope Galata, 16 June 1880, *Sintenis et Rigo 742* (P00260379; FI); Cyprus, near Phini. On dripping tufa by roadside, 5 June 1962, *R.D. Meikle 2874* (P00242688); Zypern: Trooditissa Monastery, Division 2 (sensu Meikle 1979, 1985), at the waterfall in hairpin bend E of Monastery, wet rocks, 1315 m (L: 32°50'33"E/ B: 34°54'45"N), 24 Sep. 2010, *Hand 5739* (B100342825); Insule Cypri, Nikosia, pr. le gauche a Kordukkis, 28 March 1905, *J. Holmboe 292* (O-V2262581); Insulae Cypri, Troodos: Pasha Livadia, 12 July 1905, *J. Holmboe 1075* (O-V2262581); Cedar Valley, Cedar hiking path, 34°59'28.58"N, 32°41'19.65"E, 1126 m, 7 June 2019, *S. Cambria s.n.* (CAT).

### ﻿3. Solenopsis bacchettae

Taxon classificationPlantaeAsteralesCampanulaceae

Brullo, C.Brullo, Tavilla, Siracusa & Cambria, Nord. J. Bot. 40 (12): 2, e03773.

454E5C6D-4813-57D4-B979-AFE08841B596

Figs 6F , 7E , 8H , 9F

Laurentia tenella Moris, Fl. Sardoa: 542, 1840–1843, non A. DC. Prodr. 7(2): 410, 1839.
Solenopsis bivonae auct. Flora Sarda, non M. B. Crespo, Serra & A. Juan, Pl. Syst. Evol. 210: 219, 1998.
Solenopsis minuta (L.) C. Presl subsp. minuta sensu Arrigoni, Fl. Is. Sard. 4: 532, 2013, non C. Presl (C. Presl 1836, p. 32).

#### Type.

Italy. Sardinia. Montarbu di Seui, lungo la strada sterrata ad est di Bruncu Arrascialei, su pareti umide, 986 m, 39°24'09"N, 9°53'32"E, 23 July 2018, *S. Cambria s.n.* (holotype: CAT, isotypes: CAT, CAG).

#### Description.

It differs from *S.bivonae* in having a basal rosette 3–10 cm in diameter, with leaves 12–60 mm long, hairy mainly on the blade, which is 5–25 × 2–12 mm and petiole 7–35 mm long; floral pedicels 2.5–7.5 cm, with bracteoles, in the lower half, 3–5.5 mm long, 0.4–0.5 mm wide, with 1–4 sessile glands at the margin per side; calyx (3.5)4–6.5 mm long, with lobes 2–3.5 mm long;c; corolla 13–16 mm long, uniformly dark blue–lilac, with tube blue-lilac, 5–6 mm long, 1–1.5 mm in diameter; upper lip with ovate-lanceolate lobes 5–7 mm long, 2.4–4 mm wide, obtuse and mucronate at apex, without papillae; lower lip 7–10 mm long, with a large yellowish 5–lobed macula at the base, bordered in the lobes by a triangular brown macula, with two thin white strips in the central part of the throat, rarely replaced by a white halo, lobes 5–8 × 3–5 mm, only at throat covered by dense papillae 0.1–0.2 mm long; stamen filaments 5–7 mm long, anther connate into a tube 1.4–1.7 mm long; style 6–8 mm long; capsule tuberculate, 3–4 mm long; seeds pale brown, 0.50–0.52 × 0.3–0.32 mm.

#### Iconography.

Brullo et al. (2023b), Fig. 1.

#### Phenology.

Flowering May to August, fruiting June to September.

#### Etymology.

This species is dedicated to Gianluigi Bacchetta, an active botanist from Cagliari University and an expert on the Sardinian flora.

#### Distribution and ecology.

According to Brullo et al. (2023b), *Solenopsisbacchettae* is distributed in central–east Sardinia, where it is localized on carbonatic substrates (Fig. 10). It grows exclusively on damp soils along or near small streams with fresh water at 700–1000 m a.s.l., where it is a member of a plant community rich in endemic hygrophilous species.

#### Conservation status.

This species shows a scattered distribution, currently represented by few locations, where an estimated population of around 1000 individuals occurs. Based on IUCN (2022) criteria, Brullo et al. (2023b) proposed to treat it as an endangered species (EN).

#### Additional specimens examined.

See Brullo et al. (2023b).

##### ﻿Seed micromorphology

**Figure 11.**
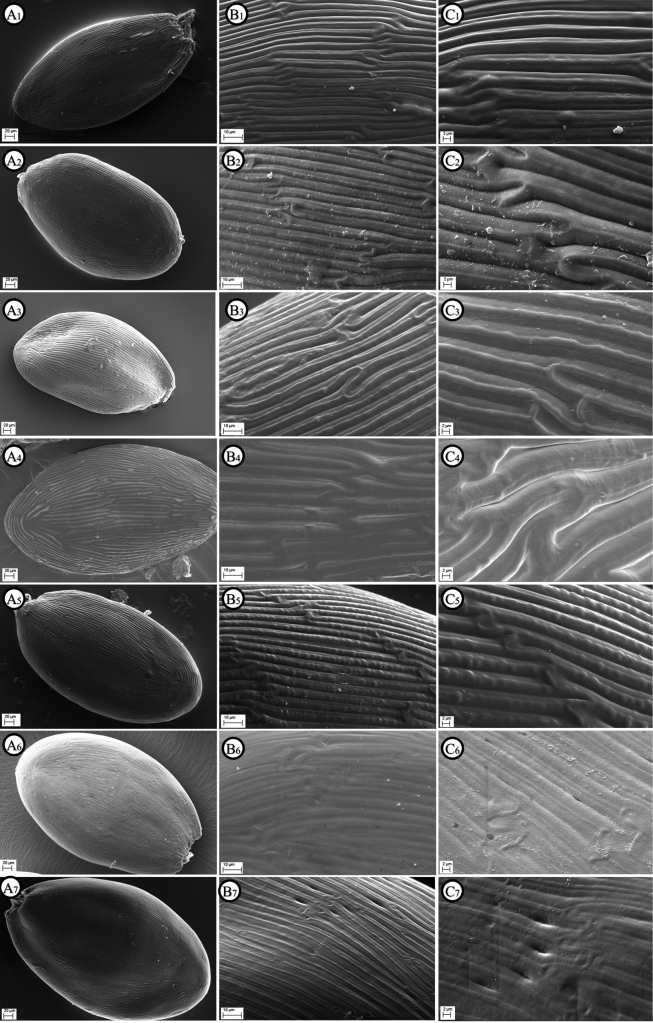
SEM images of seed shape (**A** ×250) and detail of seed testa (**B** ×1000 and **C** ×2000) regarding: **1.**Solenopsisbivonaesubsp.bivonae from Sosio river, Sicily. **2.**S.bivonaesubsp.madoniarum from Madonie massif, Sicily. **3.**S.bivonaesubsp.madoniarum from Piazza Armerina, Sicily. **4.**S.bivonaesubsp.peloritana from Mela river, Sicily. **5.**S.bivonaesubsp.brutia from Lao river, S. Italy. **6.***S.meikleana* from Cedar Valley, Cyprus. **7.***S.bacchettae* from Seui, Sardinia. Images made by Giuseppe Siracusa.

According to literature (Murata 1992, 1995; Haridasan and Mukherjee 1993; Serra and Crespo 1997; Crespo et al. 1998; Brullo et al. 2013, 2023b), the ornamentations of the seed coat in the Lobelioideae, subfamily of Campanulaceae, show a relevant taxonomical value and phylogenetic importance. Overall, the testa structure of mature seeds within this subfamily shows well-defined and constant ornamentations in every taxon. The seed coat sculptures are characterized by long, narrow cells (50–150 μm long) separated by longitudinal furrows. From the SEM observations, the seeds of Solenopsisbivonaesubsp.bivonae (Fig. 11A1) have an ellipsoid–fusiform shape, narrowing towards the basal and apical ends, having a size of 0.40–0.45 × 0.20–0.25 mm. As concerns its seed testa, the cells have periclinal walls distinctly convex, 4–5 μm wide, crossed by a marked convex central ridge 1.4–1.8 μm wide, with anticlinal walls linear and deeply grooved (Fig. 11B1–C1). The seeds of S.bivonaesubsp.madoniarum (Fig. 11A2, A3) show an obovoid–ellipsoid shape, rounded at the apical end, with a size of 0.40–0.46 × 0.24–0.26 mm. As concerns its seed testa, the cells have periclinal walls distinctly convex, 5.5–8.0 μm wide, crossed by a marked convex central ridge 0.8–1.6 μm wide, with anticlinal walls linear and deeply grooved (Fig. 11B2–C2, B3–C3). The seeds of S.bivonaesubsp.peloritana (Fig. 11A4) have an ellipsoid shape, rounded at the apical end, with a size of 0.45–0.50 × 0.24–0.26 mm. As concerns its seed testa, the cells have periclinal walls distinctly convex and smooth, 6.4–10.0 μm wide, without a central ridge, with anticlinal walls linear and deeply grooved (Fig. 11B4–C4). The seeds of S.bivonaesubsp.brutia (Fig. 11A5) have an ellipsoid shape, rounded at the apical end, with a size of 0.46–0.50 × 0.26–0.30 mm. As concerns its seed testa, the cells have periclinal walls distinctly convex, 4.4–6.0 μm wide, crossed by a marked convex central ridge 1.4–2.0 μm wide with a row of distinct tubercles and with anticlinal walls linear and deeply grooved (Fig. 11B5–C5). The seeds of *S.meikleana* (Fig. 11A6) have a broadly ellipsoid shape, rounded at the apical end, with a size of 0.40–0.46 × 0.24–0.29 mm. Regarding its seed testa, the cells have periclinal walls slightly convex, 5.0–8.3 μm wide, crossed by an evanescent convex central ridge 0.8–1.2 μm wide, and with anticlinal walls linear and slightly grooved (Fig. 11B6–C6). The seeds of *S.bacchettae* (Fig. 11A7) have an ellipsoid shape, rounded at the apical end, with a size of 0.50–0.52 × 0.30–0.32 mm. As concerns its seed testa, the cells have periclinal walls usually quite flat, 4.0–4.5 μm wide, crossed by a slightly convex central ridge 1.0–1.6 μm wide and with anticlinal walls linear and slightly grooved (Fig. 11B7–C7).

##### ﻿Phytosociological remarks

Based on our field observations during the surveys on the populations belonging to the *Solenopsisbivonae* group, it was possible to verify that they were always localized in very specialized humid habitats, limited to very circumscribed surfaces. As previously highlighted, three main habitats can be recognized, where usually the examined populations of *Solenopsis* occur. In particular, they are represented by dripping rocky walls, peat bogs, and edges of streams or springs. As concerns the wet rocky environments, the surface is usually covered by a bryophytic layer, where individuals of *Adiantum capillus–veneris* more or less densely grow. According to Deil (1995, 1996, 1998), these habitats represent conservative environments that remain very stable in time and space, unaffected by climate change in neither geological nor current climatic variation in the Mediterranean area. Besides, these wet stands host several vicarious taxa having a Tertiary origin (Deil 1995, 1996, 1998) belonging, in particular, to *Primula*, Hypericumsect.Adenosepalum, *Pinguicola*, and relictual tropical ferns, such as *Woodwardiaradicans* (L.) Sm., *Pterisvittata* L., *P.cretica* L. and *Osmundaregalis* L. Indeed, the current floristic composition of these peculiar hygrophilous communities results from evolutionary processes within the single taxa rather than recent changes in the environmental and ecological conditions. Therefore, the plant communities within which these species now grow must be considered the impoverished remains of those dating back to the Tertiary. Due to the climatic changes during the Quaternary and the recent Holocene, these phytocoenoses generally occupy much smaller areas than in the past, remaining almost constant in their floristic composition. At the same time, the taxa that characterize them have undergone significant speciation processes, always remaining linked to the same ecological context and maintaining their phytosociological role. These communities, due to their floristic set, structure, and ecological requirements, must be referred to the phytosociological class *Adiantetea capilli–veneris* Br.–Bl. in Br.–Bl., Roussine and Nègre 1952, syntaxon distributed mainly in the Mediterranean area and Western Asia (Braun–Blanquet et al. 1952; Brullo et al. 1989; Deil 1989, 1998; de Foucault 2015). Floristically, this syntaxon is differentiated mainly by the occurrence of *Adiantum capillus–veneris* L., *Samolusvalerandi* L., which grow together with several bryophytes, among them *Eucladiumverticillatum* (With.) Bruch & Schimp., *Conocephalumconicum* (L.) Dumort., *Pelliaendiviifolia* (Dicks.) Dumort., *P.epiphylla* (L.) Corda, *Scorpiurumcircinatum* Fleischer & Loeske, *Rhynchostegiellatenella* (Dicks.) Limpr. and *Eurhynchiumpraelongum* (Hedw.) Schimp. As concerns the *Solenopsis* species treated by us in this paper, most of them are closely related to these environments belonging to the *Adiantetea capillis–veneris*, which is here represented by the order *Adiantetalia capillis–veneris* Br.–Bl ex Horvatic 1934 and the alliance *Adiantion capillis veneris* Br.–Bl ex Horvatic 1934. From the syntaxonomical point of view, the *Solenopsis* species occurring in these wet environments can be considered local characteristics of five different new associations; they are: (A) *Adianto capilli–veneris*–*Solenopsietum bivonae*, (B) *Adianto capilli–veneris*–*Solenopsietum madoniari*, (C) *Adianto capilli–veneris*–*Solenopsietum peloritanae*, (D) *Adianto capilli*–*veneris–Solenopsietum brutiae*, (E) *Adianto capilli–veneris*–*Solenopsietum meikleanae*. Their floristic composition, structure, ecology, and chorology are examined for each of them, and their nomenclatural type is provided.

###### ﻿A– *Adianto capilli–veneris–Solenopsietum bivonae* ass. nov. hoc loco (Table 1, association A)

Holotypus: rel. 7, hoc loco.

Characteristic species: Solenopsisbivonaesubsp.bivonae.

Structure and ecology: This association occurs at an elevation of 10–250 m a.s.l. in the calcareous rocky walls subject to dripping by groundwater, partially covered by a bryophytic carpet mainly represented by *Eucladiumverticillatum*, *Pelliaendiviifolia*, *Rhynchostegiellatenella*, and *Scorpiurumcircinatum*. It is differentiated physiognomically by the dominance of Solenopsisbivonaesubsp.bivonae, which with its leaf rosettes covers most of these small surfaces, usually mixing with *Adiantum capillus–veneris* and *Samolusvalerandi*. The stands colonized by this vegetation are localized especially along water–courses in the cooler and shadier places.

**Table 1.** (A) *Adianto capilli-veneris-Solenopsietum bivonae*; (B) *Adianto capilli-veneris-Solenopsietum madoniari*; (C) *Adianto capilli-veneris-Solenopsietum peloritanae*; (D) *Adianto capilli-veneris-Solenopsietum brutiae*; (E) *Adianto capilli-veneris-Solenopsietum meikleanae*.

							*			*				*			*					*					
Relevè number	1	2	3	4	5	6	7	8	9	10	11	12	13	14	15	16	17	18	19	20	21	22	23	24	25	26	27
Exposure	N	N	N	N	N	N	N	N	N	N	N	N	N	N	N	N	O	O	O	S	S	S	S	S	S	S	S
Elevation (m)	230	230	230	230	230	230	230	230	40	771	771	771	1166	600	670	700	130	130	130	1200	1200	1200	1200	1200	1200	1200	1200
Surface (mq)	50	50	50	50	50	50	50	50	10	20	20	20	10	20	20	20	5	5	5	0.6	0.5	0.5	0.4	0.4	0.5	0.5	0.8
Coverage (%)	60	80	90	100	100	80	80	80	70	60	60	60	60	70	60	70	100	100	100	80	90	90	90	90	100	100	90
Association	A	A	A	A	A	A	A	A	A	B	B	B	B	C	C	C	D	D	D	E	E	E	E	E	E	E	E
Char. Association																											
Solenopsisbivonaesubsp.bivonae	3	4	4	4	3	1	3	4	1	.	.	.	.	.	.	.	.	.	.	.	.	.	.	.	.	.	.
Solenopsisbivonaesubsp.madoniarum	.	.	.	.	.	.	.	.	.	3	3	3	3	.	.	.	.	.	.	.	.	.	.	.	.	.	.
Solenopsisbivonaesubsp.peloritana	.	.	.	.	.	.	.	.	.	.	.	.	.	3	3	2	.	.	.	.	.	.	.	.	.	.	.
Solenopsisbivonaesubsp.brutia	.	.	.	.	.	.	.	.	.	.	.	.	.	.	.	.	3	3	3	.	.	.	.	.	.	.	.
*Solenopsismeikleana*	.	.	.	.	.	.	.	.	.	.	.	.	.	.	.	.	.	.	.	2	1	3	3	2	3	2	2
*Carextroodi* Turrill	.	.	.	.	.	.	.	.	.	.	.	.	.	.	.	.	.	.	.	1	2	+	1	1	.	1	.
Char. All. (*Adiantion capilli-veneris*) and Cl. (*Adiantetea capilli-veneris*)																											
*Adiantum capillus-veneris* L.	2	1	3	4	4	4	3	2	3	1	.	+	2	2	1	2	3	2	3	1	1	1	+	+	.	1	1
*Eucladiumverticillatum* (With.) Bruch & Schimp.	.	1	.	.	.	+	+	1	2	2	2	1	1	.	.	+	2	2	2	3	2	2	3	3	3	1	2
*Samolusvalerandi* L.	+	.	1	.	+	1	+	.	+	1	1	1	+	2	1	2	.	.	.	+	.	+	+	.	+	.	+
*Pelliaepiphylla* (L.) Corda	.	.	.	.	.	.	.	.	.	.	.	.	.	2	+	1	2	2	1	2	4	2	1	2	3	4	3
*Pelliaendiviifolia* (Dicks.) Dumort.	1	1	+	+	1	+	1	.	.	.	+	1	+	.	+	.	.	.	.	.	.	.	.	.	.	.	.
*Conocephalumconicum* (L.) Dumort.	.	.	.	.	.	.	.	.	.	.	.	.	.	1	+	1	1	+	.	.	.	.	.	.	.	.	.
Other species																											
*Eurhynchiumpraelongum* (Hedw.) Schimp.	.	.	.	.	.	.	.	.	.	.	.	.	.	.	.	.	2	2	1	1	2	2	3	2	2	1	3
*Scorpiurumcircinatum* Fleischer & Loeske	+	.	.	.	+	.	+	+	.	.	.	.	.	.	.	.	.	.	.	+	1	+	+	.	+	.	.
*Rhynchostegiellatenella* (Dicks.) Limpr.	+	+	.	.	+	+	+	+	.	.	.	.	.	.	.	.	.	.	.	.	+	1	.	.	1	.	.
*Hypericumhircinum* L.	.	.	.	.	.	+	+	.	.	.	.	.	.	+	+	+	.	.	.	.	.	.	.	.	.	.	.
*Eupatoriumcannabinum* L.	.	.	.	+	.	+	+	.	+	.	.	.	.	+	+	.	.	.	.	.	.	.	.	.	.	.	.
*Lotustenuis* Waldst. & Kit. ex Willd.	.	+	1	1	.	.	.	.	.	.	.	.	.	.	.	.	.	.	.	.	.	.	.	.	.	.	.
*Equisetumarvense* L.	+	+	1	.	.	.	.	.	.	.	.	.	.	.	.	.	.	.	.	.	.	.	.	.	.	.	.
*Bryum* sp.	.	.	.	.	.	.	.	.	.	.	.	.	.	.	.	.	.	.	.	+	.	+	+	.	.	.	.
*Menthapulegium* L.	.	.	.	+	+	+	.	.	.	.	.	.	.	.	.	.	.	.	.	.	.	.	.	.	.	.	.
*Crepisleontodontoides* All.	.	.	.	+	+	.	.	.	.	.	.	.	.	.	.	.	.	.	.	.	.	.	.	.	.	.	.
*Pulicariadysenterica* (L.) Bernh.	.	.	.	.	.	.	+	+	.	.	.	.	.	.	.	.	.	.	.	.	.	.	.	.	.	.	.
*Lysimachianemorum* L.	.	.	.	.	.	.	.	.	.	.	.	.	.	+	1	.	.	.	.	.	.	.	.	.	.	.	.
*Agrostisstolonifera* L.	.	.	.	.	.	.	.	.	.	.	.	.	.	.	+	+	.	.	.	.	.	.	.	.	.	.	.
*Mycelismuralis* (L.) Dumort.	.	.	.	.	.	.	.	.	.	.	.	.	.	.	+	+	.	.	.	.	.	.	.	.	.	.	.
*Brachypodiumsylvaticum* (Huds.) P.Beauv.	.	.	.	.	.	.	.	.	.	.	.	.	.	.	+	+	.	.	.	.	.	.	.	.	.	.	.
*Fissidenstaxifolius* Hedw.	.	.	.	.	.	.	.	.	.	.	.	.	.	.	+	1	.	.	.	.	.	.	.	.	.	.	.
*Carex* sp.	.	.	1	.	+	.	.	.	.	.	.	.	.	.	.	.	.	.	.	.	.	.	.	.	.	.	.
*Potentillareptans* L.	.	.	.	.	.	.	.	.	.	.	.	.	.	.	.	.	+	.	1	.	.	.	.	.	.	.	.
*Centauriumpulchellum* (Sw.) Druce	.	.	+	.	.	.	.	.	.	.	.	.	.	.	.	.	.	.	.	.	.	.	.	.	.	.	.
Dittrichiaviscosa(L.)Greutersubsp.viscosa	+	.	.	.	.	.	.	.	.	.	.	.	.	.	.	.	.	.	.	.	.	.	.	.	.	.	.
*Chenopodiumalbum* L.	.	+	.	.	.	.	.	.	.	.	.	.	.	.	.	.	.	.	.	.	.	.	.	.	.	.	.
*Carexpendula* Huds.	.	.	.	.	.	.	.	.	.	.	.	.	.	.	+	.	.	.	.	.	.	.	.	.	.	.	.
*Angelicasylvestris* L.	.	.	.	.	.	.	.	.	.	.	.	.	.	.	+	.	.	.	.	.	.	.	.	.	.	.	.
*Helosciadiumnodiflorum* (L.) W.D.J.Koch	.	.	.	.	.	.	.	.	.	.	.	.	.	.	+	.	.	.	.	.	.	.	.	.	.	.	.
ViolaalbaBessersubsp.dehnhardtii (Ten.) W.Becker	.	.	.	.	.	.	.	.	.	.	.	.	.	.	.	+	.	.	.	.	.	.	.	.	.	.	.
*Hypericumtetrapterum* Fr.	.	.	.	.	.	.	.	.	.	+	.	.	.	.	.	.	.	.	.	.	.	.	.	.	.	.	.

Rel. 1–8: Sosio River (Chiusa Sclafani), 10/07/2018 Rel. 9: Oreto River (Palermo), 29/07/2018 Rel. 10–12: Mt. Canalotto (Piazza Armerina), 16/10/2021 Rel. 13: Cataratta Spring (Petralia Soprana (15/07/2017 Rel. 14–16: Passo Pirtuso Valley, S. Lucia del Mela, 19/7/2020 Rel. 17–19: Lao River, Papasidero (Calabria), 6/08/2018 Rel. 20–27: Caledonia Waterfalls, Cyprus 06/10/1988

Distribution: The association was surveyed along the Sosio river near Chiusa Sclafani, where it is quite frequent, and the Oreto River near Palermo, where, however, it is currently very rare.

###### ﻿B– *Adianto capilli–veneris*–*Solenopsietum madoniari* ass. nov. hoc loco (Table 1, association B)

Holotypus: rel. 10, hoc loco.

Characteristic species: Solenopsisbivonaesubsp.madoniarum.

Structure and ecology: This association is localized in a habitat very similar to those colonized by the previous one but linked to stands with higher elevation (700–1200 m a.s.l.). This vegetation shows a lower coverage of *Adiantum capillus–veneris* and a more developed bryophytic layer, characterized by *Eucladiumverticillatum* and *Pelliaendiviifolia*. This habitat is represented by vertical rocky walls with dripping waters coming from small springs.

Distribution: This association is quite rare, and was observed in a few mountain localities, like near Piazza Armerian and Petralia Soprana.

###### ﻿C– *Adianto capilli–veneris*–*Solenopsietum peloritanae* ass. nov. hoc loco (Table 1, association C)

Holotypus: rel. 14, hoc loco.

Characteristic species: Solenopsisbivonaesubsp.peloritana.

Structure and ecology: It is a sub-mountain association closely linked to metamorphic vertical rocky walls with dripping groundwaters at an elevation of 600–700 m a.s.l. The bryophytic layer is represented by *Pelliaepiphylla* and *Conocephalumconicum*, where *Adiantum capillus–veneris*, Solenopsisbivonaesubsp.peloritana and *Samolusvalerandi* grow, often with high values of coverage.

Distribution: This association is exclusive of a small stand in the Tyrrhenian slope of the Peloritani range.

###### ﻿D– *Adianto capilli–veneris*–*Solenopsietum brutiae* ass. nov. hoc loco (Table 1, association D)

Holotypus: rel. 17, hoc loco.

Characteristic species: Solenopsisbivonaesubsp.brutia.

Structure and ecology: This association was surveyed on metamorphic wet rocky outcrops along the banks of perennial water–courses at an elevation of 130–350 m a.s.l. Physiognomically, this vegetation is dominated by *Adiantum capillus–veneris* and Solenopsisbivonaesubsp.brutia, which grow on a well-developed bryophytic layer, characterized by *Pelliaepiphylla*, *Eucladiumverticillatum*, *Conocephalumconicum*, and *Eurhynchiumpraelongum*.

Distribution: This association was observed in North Calabria, along the banks of the lower reaches of the Lao river.

###### ﻿E– *Adianto capilli–veneris*–*Solenopsietummeikleanae* ass. nov. hoc loco (Table 1, association E)

Holotypus: rel 22, hoc loco.

Characteristic species: *Solenopsismeikleana* and *Carextroodi* Turril.

Structure and ecology: This association usually is linked to wetlands represented mainly by waterfalls and dripping walls, often near the spring, where it grows on ophiolitic substrata at an elevation of 1000–1600 m a.s.l. The vegetation is localized prevalently in the stands not directly affected by the water flow, liking less damp surfaces. In the bryophytic layer, the more frequent species are *Eucladiumverticillatum*, *Pelliaepiphylla*, *Eurhynchiumpraelongum*, and *Scorpiurumcircinatum*, while among the vascular plants, the endemic *Solenopsismeikleana* and *Carextroodi* are dominant, growing together with *Adiantum capillus–veneris*.

Distribution: This association is endemic to the western part of the island of Cyprus, which is localized in very specialized damp habitats.

As concerns Solenopsisbivonaesubsp.madoniarum, in Sicily it is more widespread in the peatlands, an uncommon and peculiar habitat, currently exclusive of the mountain belt of Madonie massif at an elevation of 1200–1600 m a.s.l. In this area, the bog mosses dominated by *Sphagnum* sp. pl. are circumscribed to small surfaces with groundwater emerging or fed by springs, limitedly to highly acidic substrates with siliceous origin. These stands, locally known as tremulous lands, host a very specialized vegetation dominated by a thick and deep layer of *Sphagnum* species, which is here represented mainly by *S.auriculatum* Schimp. and *S.inundatum* Russow [= *S.obesum* (Wilson) Warnst], which are associated with *Aulacomniumpalustre* (Hedw.) Swaegr., *Polytrichumcommune* Hedw., *Bryumpseudotriquetrum* (Hedw.) P. Gaertn. et al., *Philonotisfontana* (Hedw.) Brid., *Callirgoniellacuspidata* (Hedw.) Loeske, etc. (Raimondo and Dia 1978; Raimondo et al. 2021). The phytosociological relevés carried out by some of the authors, always on the Madonie massif (Table 2), agree quite well with those previously published by Petronici et al. (1978) and Raimondo et al. (1980, 2021). This vegetation, where Solenopsisbivonaesubsp.madoniarum grows together with the bryophytes mentioned above, was attributed by Raimondo et al. (2021) to a new association proposed as *Sphagnoauriculati–Caricetumechinatae* and arranged in the *Caricionfuscae* Koch, 1926, an alliance of the *Scheuchzeriopalustris–Cariceteafuscae* Tüxen, 1937. This class is distributed in the Euro–Siberian territory, reaching the Mediterranean region, limited to restricted mountain stands, which assume a relict meaning.

**Table 2.** *Sphagnoauriculati*-*Caricetumechinatae*.

Relevè number	1	2	3	4	5	6	7	8	9	10
Elevation (dam)	138	138	138	138	138	138	140	140	140	140
Surface (mq)	5	5	2	4	4	4	5	2	2	4
Coverage (%)	100	100	100	100	100	100	100	100	100	100
Char. Association										
Solenonpsisbivonaesubsp.madoniarum	1	2	3	3	2	1	2	1	3	1
*Carexpaniculata* L.	.	.	+	.	1	1	+	+	+	.
Char. All. (*Caricionnigrae*) and Ord. (*Caricetalianigrae*)										
*Aulacomniumpalustre* (Hedw.) Swaegr.	1	2	+	+	2	1	1	3	2	2
*Carexpunctata* Gaudin	+	+	.	1	1	1	1	.	.	+
Char. Cl. (*Scheuchzerio*-*Caricetea nigrae*)										
*Sphagnuminundatum* Russow	3	3	2	2	3	4	3	3	3	4
*Carexechinata* Murray	4	2	3	3	1	3	1	2	2	2
*Carexdemissa* Hornem.	+	.	1	+	+	.	+	.	+	.
*Polytrichumcommune* Hedw.	2	1	.	.	2	.	.	1	1	.
*Deschampsiacaespitosa* (L.) P.Beauv.	.	.	.	1	1	.	.	.	.	.
Other species										
*Juncusfontanesii* J. Gay	3	5	3	2	4	3	4	4	4	3
*Poatrivialis* L.	1	1	+	1	+	1	1	1	+	1
*Menthaaquatica* L.	+	1	+	2	1	1	2	3	2	1
*Holcuslanatus* L.	2	2	1	2	1	2	2	2	1	1
*Juncusconglomeratus* L.	1	2	3	2	2	1	3	2	2	1
*Festucacircumediterranea* Patzke	2	2	2	1	1	2	+	.	+	1
*Juncusstriatus* Schousb. ex E.Mey.	.	1	1	+	+	2	1	1	2	1
*Bryumpseudotriquetrum* (Hedw.) P. Gaertn. et al.	1	.	1	1	1	1	1	1	1	+
*Hypericumtetrapterum* Fr.	.	.	.	2	1	+	1	2	2	1
Dactylorhizamaculata(L.)Soósubsp.saccifera (Brongn.) Diklić	1	2	2	2	2	2	.	.	.	.
*Carexremota* L.	.	+	.	+	.	1	.	+	.	+
*Carexovalis* Gooden	.	.	.	.	.	1	1	+	+	+
*Bellishybrida* Ten.	.	1	1	1	1	+	.	.	.	.
*Dactylisglomerata* L.	.	.	.	.	.	.	2	2	2	1
*Isolepissetacea* (L.) R.Br.	.	.	.	.	.	.	1	2	1	1
*Trifoliumrepens* L.	.	.	.	.	.	.	+	1	+	+
*Philonotisfontana* (Hedw.) Brid.	.	.	.	.	.	.	2	2	2	1
*Ranunculusfontanus* C. Presl.	.	.	.	.	.	.	+	+	1	.
Cirsiumcreticum(Lam.)d‘Urv.subsp.triumfettii (Lacaita) K.Werner	.	+	.	+	+	.	.	.	.	.
*Pulicariadysenterica* (L.) Bernh.	.	.	.	2	1	1	.	.	.	.
*Jungermanniagracillima* Sm.	1	.	+	.	.	1	.	.	.	.
*Lycopuseuropaeus* L.	.	.	.	.	.	.	+	2	1	.
*Utriculariaaustralis* R.Br.	1	.	+	.	.	1	.	.	.	.
*Cynosuruscristatus* L.	.	.	.	.	.	.	1	1	1	.
GaliumpalustreL.subsp.elongatum (C. Presl) Arcang.	.	.	.	.	.	.	+	.	+	.
*Bechnumspicant* (L.) Sm.	+	.	.	.	.	+	.	.	.	.
*Lysimachianemorum* L.	.	.	.	.	2	1	.	.	.	.

Rel. 1–10, Portella Mandarini, Madonie, 31.7.1990

### ﻿Key to the taxa belonging to the *Solenopsisbivonae* group

Basing on the morphological diacritical characters listed in Table 3, the following analytical key has been performed.

**Table 3.** Diagnostic characters of taxa belonging to *Solenopsisbivonae* group.

Taxa	S.bivonaesubsp.bivonae	S.bivonaesubsp.madoniarum	S.bivonaesubsp.peloritana	S.bivonaesubsp.brutia	*S.meikleana*	*S.bacchettae*
Characters						
Leaf rosula diameter (cm)	2–12.5	3.5–8	4–10	2.5–7	2.5–11	3–10
Occurence of stolons	no	no	no	no	yes	no
Leaf indumentum	glabrous	glabrous	glabrous	glabrous	glabrous to hairy	hairy
Leaf shape	spathulate	oblanceolate–spathulate	spathulate	oblanceolate–spathulate	oblanceolate–spathulate	oblanceolate–spathulate
Leaf length (mm)	12–100	15–45	12–55	10–58	10–75	12–60
Leaf petiole length (mm)	5–60	8–25	5–30	3–36	5–50	7–35
Leaf blade size (mm)	6–40 × 4–15	4–20 × 2–8	7–23 × 4–10	5–22 × 2–10	6–30 × 4–15	5–25 × 2–12
Floral pedicel lenght (mm)	5–11	2–5(9)	5.5–11	3–6	2–12	2.5–7.5
Bracteole number	1(2)	1	2	2	1–2	1–2
Bracteole size (mm)	2–2.4 × 0.3–0.5	1.8–2.2 × 0.1–0.3	3–5.5 × 0.4–0.7	2–3 × 0.25–0.45	2–8 × 0.2–0.6	3–5.5 × 0.4–0.5
Bracteole apex	hairy	few hairs	glabrous with one gland	glabrous with one gland	glabrous with one gland	hairy
Bracteole lateral glands	1–4	1–2	1–2	1–3	1–2	1–4
Calyx lenght (mm)	3–4	3–4	4–5	3.5–5	3.5	(3.5)4–6.5
Calyx lobes lenght (mm)	2–3	2–3.5	3.2–4	2–3	1.5–3	2–3.5
Corolla lenght (mm)	10–12	8.5–10	12–14.5	11–12	10–12	13–16
Corolla tube lenght (mm)	4–5	3.7–4.5	3.5–4	4.5–5	3–5	5–6
Corolla tube diameter (mm)	ca. 1	0.9–1.3	ca. 1.5	1–1.2	1.1–1.3	1–1.5
Corolla tube colour	lilac	lilac	green	white–lilac	green–violet	blue–lilac
Corolla upper lip shape	linear–lanceolate	linear–lanceolate	linear–lanceolate	linear–lanceolate	linear–lanceolate	ovate–lanceolate
Corolla upper lip size (mm)	3.5–4.5 × 1.3–1.7	3–4 × 1.2–1.7	5–6 × 2–2.4	4–4.5 × 1.4–1.8	3.5–4.5 × 1.5–1.7	5–7 × 2.4–4
Corolla upper lip papillae	yes	no	yes	yes	no	no
Corolla upper lip colour	bluish–lilac	bluish–lilac	dark lilac	bluish–lilac	pale blue to pale violet	dark blue–lilac
Corolla upper lip apex	acute	obtuse	acute	subobtuse	acute	obtuse
Corolla lower lip lenght (mm)	5–7	5–6	8–9	6.5–7	5–5.5	7–10
Corolla lower lip colour	bluish–lilac, white in central part	bluish–lilac, white in central part	bluish–lilac, white in central part	bluish–lilac, white in central part	pale blue to pale violet, white in central part	uniformely dark blue–lilac, rarely with a basal white alone
Corolla lower lip macula	greenish–yellow bordered of brown at base	yellowish, bordered of brown at base	yellow, bordered of red–brown above, with a central red–brown spot	greenish, with three distinct dark blue spots, bordered of brown	greenish–yellow	yellowish, 5 lobed, bordered of brown
Lobes of lower lip shape	ovate and mucronate	ovate and obtuse	obovate and mucronate	ovate and mucronate	oblong–obtuse, mucronate	widely ovate, mucronate
Lobes of lower lip size (mm)	3.5–4.5 × 3–4	2.5–3.5 × 1.6–2.5	4.5–6.5 × 4–4.5	3.5–5 × 2.5–3.5	2.5–3.5 × 1.4–2.2	5–8 × 3–5
Papillae of lower lip lobes	covering more than lower half	covering the lower half	covering almost until the apex	covering almost until the apex	covering more than lower half	covering only the throat
Papillae density	not very dense	very dense	very dense	not very dense	not very dense	very dense
Papillae lenght	0.25–0.6	0.1–0.24	0.1–0.4	0.16–0.6	0.05–0.3	0.1–0.2
Staminal filament lenght (mm)	4–4.5	4–4.5	4.5–4.7	5–5.5	3–5	5–7
Anther tube lenght (mm)	1.5–1.8	1.4–1.6	1.7–1.9	1.5–1.6	1–1.5	1.4–1.7
Anther tube basal papillae	no	yes	no	no	yes	no
Anther tube dorsal hairiness	yes	yes	yes	yes	yes	yes
Style lenght (mm)	4–4.5	4.5–5.5	6.5–7	6–6.5	3.5–4	6–8
Capsule lenght (mm)	1.6–2	2.7–3	2.5–3	2–3.3	3–3.2	3–4
Capsule surface	smooth	smooth	tubercolate	tubercolate	smooth	tuberculate
Seeds size (mm)	0.40–0.45 × 0.20–0.25	0.40–0.46 × 0.24–0.26	0.44–0.50 × 0.24–0.26	0.46–0.50 × 0.26–0.30	0.40–0.46 × 0.24–0.29	0.5–0.52 × 0.3–0.32

**Table:** 

1	Leaves always hairy; corolla uniformly dark blue–lilac, with upper lip ovate–lanceolate, 2.4–4 mm wide; papillae localized only in the throat	***S.bacchettae***
–	Leaves glabrous, rarely subglabrous; corolla pale blue–violet to bluish-lilac with lower lip white in the basal part, with upper lip linear–lanceolate, 1.2–2.4 wide; papillae spread along the lips	**2**
2	Bracteoles 2–8 mm long; corolla tube green–violet; corolla lips pale blue to pale violet; corolla throat uniformly greenish–yellow; corolla lower lip with lobes oblong, anther tube 1–1.5 mm long; style 3.5–4 mm long	***S.meikleana* sp. nov.**
–	Bracteoles 1.8–2.5 mm long; corolla tube white–lilac to lilac; corolla lips bluish–lilac; corolla throat yellowish to greenish bordered of brown; corolla lower lip with lobes ovate; anther tube 1.4–1.9 mm long.; style 4–7 mm long	**3**
3	Bracteoles 3–5.5 mm long; corolla 12–14.5 mm long, with upper lip 5–6 mm long and lower lip 8–9 mm long; style 6.5–7 mm long	**S.bivonaesubsp.peloritana**
–	Bracteoles 1.8–3 mm long; corolla 8.5–12 mm long, with upper lip 3–4.5 mm long and lower lip 5–7 mm long; style 4–6.5 mm long	**4**
4	Floral pedicel with one bracteole; corolla with lobes of the upper lip without papillae and lobes of lower lip 2.5–3.5 mm long and 1.6–2.5 mm wide; papillae up to 0.24 mm long; anther tube provided by basal papillae	**S.bivonaesubsp.madoniarum**
–	Floral pedicel with two bracteoles or rarely with one bracteole; corolla with lobes of upper lip partially covered by papillae and lobes of lower lip 3.5–5 mm long and 2.5–4 mm wide; papillae up to 0.6 mm long; anther tube without basal papillae	**5**
5	Floral pedicel 3–6 mm long; bracteoles glabrous at the apex; lower lip of corolla with macula bordered with three distinct dark blue spots; staminal filament 5–5.5 mm long; style 6–6.5; capsule 2–3.3 mm long	**S.bivonaesubsp.brutia**
–	Floral pedicel 5–11 mm long; bracteoles hairy at the apex; lower lip of corolla with macula without dark spots; staminal filament 4–4.5 mm long; style 4–4.5, capsule 1.6–2 mm long	**S.bivonaesubsp.bivonae**

## Supplementary Material

XML Treatment for Solenopsis bivonae

XML Treatment for Solenopsis bivonae

XML Treatment for Solenopsis bivonae

XML Treatment for Solenopsis bivonae

XML Treatment for Solenopsis bivonae

XML Treatment for Solenopsis meikleana

XML Treatment for Solenopsis bacchettae

## Additional information

### Author ORCIDs

Salvatore Brullo https://orcid.org/0000-0003-2568-7278

Salvatore Cambria https://orcid.org/0000-0002-3828-1552

Valeria Tomaselli https://orcid.org/0000-0001-9121-9558

Alessandro Crisafulli https://orcid.org/0000-0003-4398-345X

Pietro Minissale https://orcid.org/0000-0002-4047-4169

Gianpietro Giusso del Galdo https://orcid.org/0000-0003-4719-3711

### Data availability

All of the data that support the findings of this study are available in the main text or Supplementary Information.
